# Supplementary material for: The efficacy of promoting sustained shared thinking through the use of activity books on parental empowerment; A quasi-experimental study
Source: PLoS One. 2025 Jul 18;20(7):e0328537. doi: 10.1371/journal.pone.0328537 (PMC12273987; doi:10.1371/journal.pone.0328537)
Supplement: S1 Dataset — (ZIP) [file pone.0328537.s001.zip › S1 Dataset/Dataset1/Output CFA.pdf]

```
. do "C:\Users\66863\AppData\Local\Temp\STD419c_000000.tmp"

. sem (INTRAPERSONAL -> PE1, ) (INTRAPERSONAL -> PE2, ) (INTRAPERSONAL -> PE3, ) (INTRAPERSONAL -> PE4, ) ///
>     (INTERACTIONAL -> PE5, ) (INTERACTIONAL -> PE6, ) (INTERACTIONAL -> PE7, ) (INTERACTIONAL -> PE8, ) (INTERACTIONAL
>  ) ///
>     (BEHAVIORAL -> PE10, ) (BEHAVIORAL -> PE11, ) (BEHAVIORAL -> PE12, ) ///
>     , covstruct(_lexogenous, diagonal) standardized latent(INTRAPERSONAL INTERACTIONAL BEHAVIORAL ) ///
>     cov( INTRAPERSONAL*INTERACTIONAL INTRAPERSONAL*BEHAVIORAL e.PE1*e.PE6 e.PE4*e.PE5 INTERACTIONAL*BEHAVIORAL e.PE5*e
> E6*e.PE8 e.PE7*e.PE8 e.PE10*e.PE11) nocapslatent

Endogenous variables
  Measurement: PE1 PE2 PE3 PE4 PE5 PE6 PE7 PE8 PE9 PE10 PE11 PE12

Exogenous variables
  Latent: INTRAPERSONAL INTERACTIONAL BEHAVIORAL

Fitting target model:
Iteration 0:   log likelihood = -1513.5182  (not concave)
Iteration 1:   log likelihood = -1478.2921  (not concave)
Iteration 2:   log likelihood = -1466.7697  (not concave)
Iteration 3:   log likelihood = -1459.0675  (not concave)
Iteration 4:   log likelihood =  -1456.869  (not concave)
Iteration 5:   log likelihood = -1454.2458  (not concave)
Iteration 6:   log likelihood = -1452.9896  (not concave)
Iteration 7:   log likelihood = -1452.1304  (not concave)
Iteration 8:   log likelihood = -1451.2716  (not concave)
Iteration 9:   log likelihood = -1449.6117  (not concave)
Iteration 10:  log likelihood =  -1448.748
Iteration 11:  log likelihood = -1447.5045
Iteration 12:  log likelihood = -1446.6926
Iteration 13:  log likelihood = -1445.5312
Iteration 14:  log likelihood = -1445.3344
Iteration 15:  log likelihood = -1445.1969
Iteration 16:  log likelihood = -1445.1476
Iteration 17:  log likelihood = -1445.1309
Iteration 18:  log likelihood = -1445.1301
Iteration 19:  log likelihood = -1445.1301

Structural equation model                            Number of obs = 160
Estimation method: ml

Log likelihood = -1445.1301

 ( 1)  [PE1]INTRAPERSONAL = 1
 ( 2)  [PE5]INTERACTIONAL = 1
 ( 3)  [PE10]BEHAVIORAL = 1
```

Standardized	Coefficient	OIM std. err.	z	P>|z|	[95% conf.	interval]
**Measurement**						
PE1						
INTRAPERSONAL	.1500808	.0949233	1.58	0.114	-.0359655	.3361272
_cons	13.46099	.7533688	17.87	0.000	11.98442	14.93757
PE2						
INTRAPERSONAL	.6640012	.0717094	9.26	0.000	.5234533	.804549
_cons	6.121546	.3512144	17.43	0.000	5.433178	6.809913
PE3						
INTRAPERSONAL	.5486704	.0770256	7.12	0.000	.3977029	.6996378
_cons	9.708607	.5484509	17.70	0.000	8.633663	10.78355
PE4						
INTRAPERSONAL	.590204	.0744631	7.93	0.000	.4442589	.736149
_cons	9.079541	.5120432	17.73	0.000	8.075955	10.08313

PE5						
INTERACTIONAL	.4775485	.0698282	6.84	0.000	.3406876	.6144093
_cons	8.642115	.4864473	17.77	0.000	7.688696	9.595534
PE6						
INTERACTIONAL	.1931879	.0886213	2.18	0.029	.0194934	.3668824
_cons	15.9225	.8882568	17.93	0.000	14.18155	17.66345
PE7						
INTERACTIONAL	.7606774	.0615626	12.36	0.000	.6400169	.8813379
_cons	7.089791	.4041391	17.54	0.000	6.297693	7.881889
PE8						
INTERACTIONAL	.712914	.0662645	10.76	0.000	.5830379	.84279
_cons	6.872604	.3916881	17.55	0.000	6.10491	7.640299
PE9						
INTERACTIONAL	.6166545	.0625995	9.85	0.000	.4939617	.7393473
_cons	6.539564	.3740233	17.48	0.000	5.806492	7.272637
PE10						
BEHAVIORAL	.5271485	.1290326	4.09	0.000	.2742493	.7800477
_cons	4.986707	.2897588	17.21	0.000	4.41879	5.554624
PE11						
BEHAVIORAL	.3560622	.104608	3.40	0.001	.1510344	.5610901
_cons	5.33102	.3083209	17.29	0.000	4.726722	5.935318
PE12						
BEHAVIORAL	.2983883	.096979	3.08	0.002	.108313	.4884636
_cons	5.072776	.2943906	17.23	0.000	4.495781	5.649771
var(e.PE1)	.9774757	.0284923			.923197	1.034946
var(e.PE2)	.5591024	.0952303			.4004135	.7806818
var(e.PE3)	.6989608	.0845233			.5514669	.8859031
var(e.PE4)	.6516592	.0878969			.5002754	.8488521
var(e.PE5)	.7719474	.0666927			.6517001	.914382
var(e.PE6)	.9626784	.0342411			.8978529	1.032184
var(e.PE7)	.4213699	.0936586			.2725621	.6514206
var(e.PE8)	.4917537	.0944818			.337446	.7166234
var(e.PE9)	.6197372	.0772045			.4854763	.7911286
var(e.PE10)	.7221145	.1360387			.4991704	1.044632
var(e.PE11)	.8732197	.0744939			.7387678	1.032141
var(e.PE12)	.9109644	.0578748			.80431	1.031762
var(INTRAPERSONAL)	1	.			.	.
var(INTERACTIONAL)	1	.			.	.
var(BEHAVIORAL)	1	.			.	.
cov(e.PE1,e.PE6)	.2267206	.0761604	2.98	0.003	.0774489	.3759922
cov(e.PE4,e.PE5)	.2855838	.0812443	3.52	0.000	.126348	.4448196
cov(e.PE5,e.PE6)	.1291265	.0768222	1.68	0.093	-.0214422	.2796952
cov(e.PE6,e.PE8)	-.1542707	.0980734	-1.57	0.116	-.3464911	.0379496
cov(e.PE7,e.PE8)	-.3240156	.1874971	-1.73	0.084	-.6915031	.0434719
cov(e.PE10,e.PE11)	.4942308	.0752356	6.57	0.000	.3467716	.6416899
cov(INTRAPERSONAL,INTERACTIONAL)	.6735183	.0812621	8.29	0.000	.5142476	.832789
cov(INTRAPERSONAL,BEHAVIORAL)	.6984358	.2062616	3.39	0.001	.2941705	1.102701
cov(INTERACTIONAL,BEHAVIORAL)	.9695732	.2102086	4.61	0.000	.5575718	1.381575

LR test of model vs. saturated: chi2(45) = 48.52 Prob > chi2 = 0.3330

```
.
end of do-file

. do "C:\Users\66863\AppData\Local\Temp\STD419c_000000.tmp"

. estat gof, stats(all)
```

Fit statistic	Value	Description
Likelihood ratio		
chi2_ms(**45**)	**48.519**	model vs. saturated
p > chi2	**0.333**	
chi2_bs(**66**)	**419.118**	baseline vs. saturated
p > chi2	**0.000**	
Population error		
RMSEA	**0.022**	Root mean squared error of approximation
90% CI, lower bound	**0.000**	
upper bound	**0.059**	
pclose	**0.877**	Probability RMSEA <= 0.05
Information criteria		
AIC	**2980.260**	Akaike's information criterion
BIC	**3118.643**	Bayesian information criterion
Baseline comparison		
CFI	**0.990**	Comparative fit index
TLI	**0.985**	Tucker-Lewis index
Size of residuals		
SRMR	**0.056**	Standardized root mean squared residual
CD	**0.924**	Coefficient of determination

```
.
end of do-file

.
```
