# Supplementary figures and images for: The efficacy of promoting sustained shared thinking through the use of activity books on parental empowerment; A quasi-experimental study
Source: PLoS One. 2025 Jul 18;20(7):e0328537. doi: 10.1371/journal.pone.0328537 (PMC12273987; doi:10.1371/journal.pone.0328537)

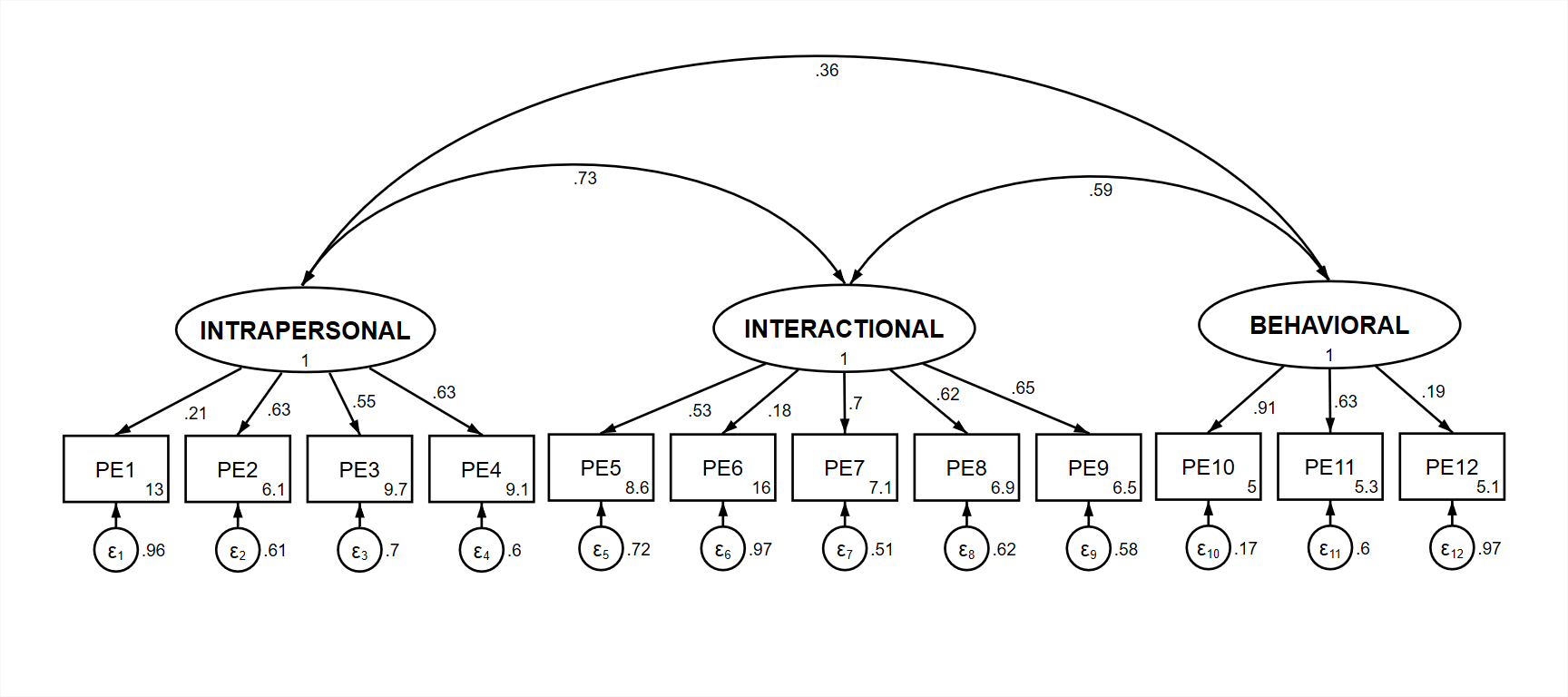

Supplement: S1 Dataset — (ZIP) [file pone.0328537.s001.zip › S1 Dataset/Dataset1/CFA (model 1).tif]

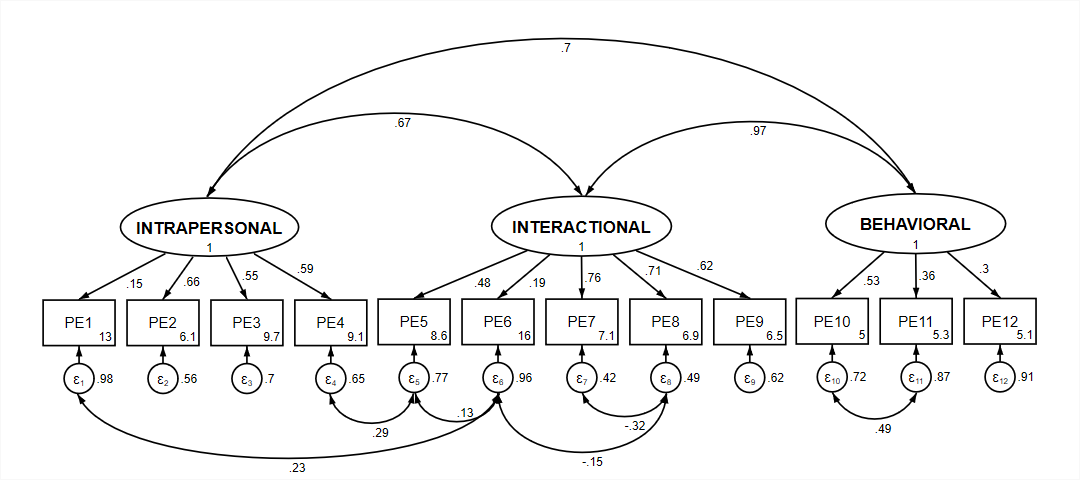

Supplement: S1 Dataset — (ZIP) [file pone.0328537.s001.zip › S1 Dataset/Dataset1/CFA (model final).tif]

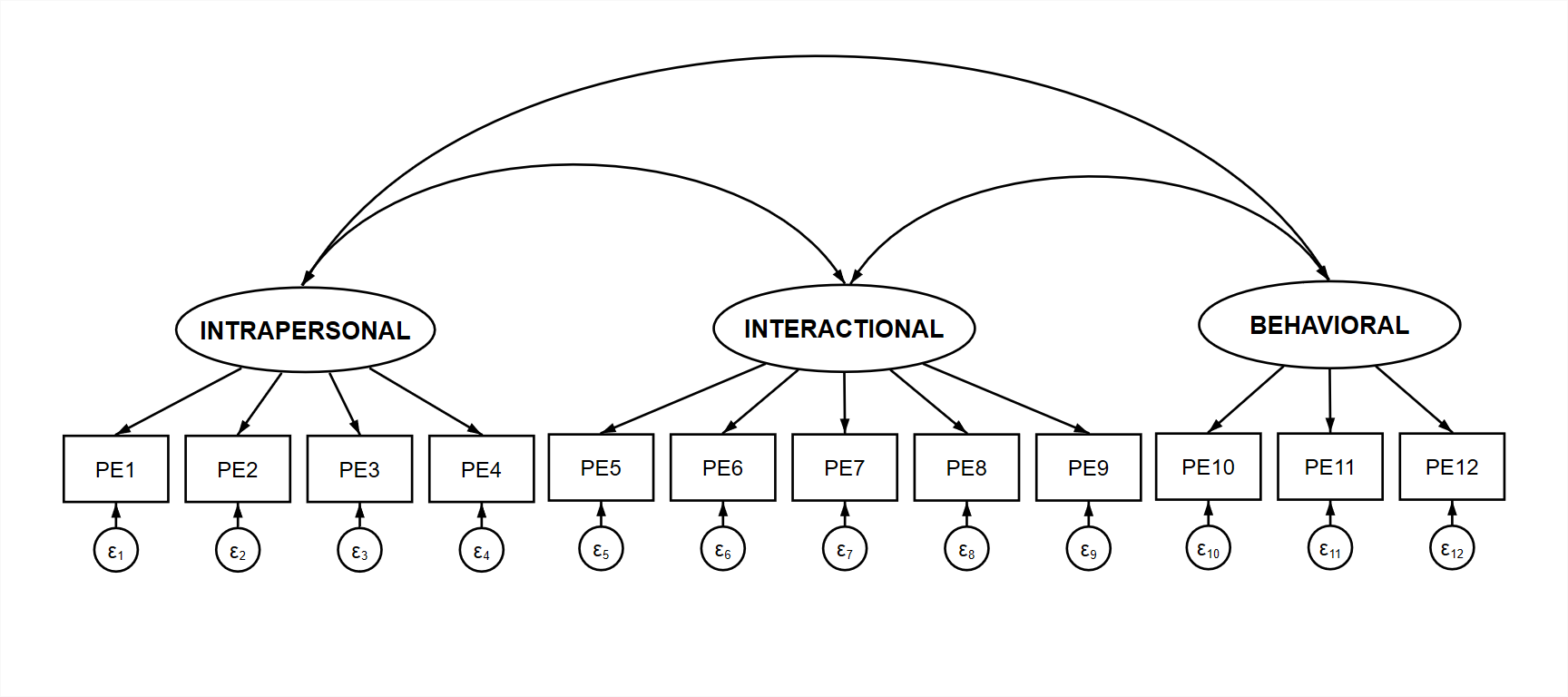

Supplement: S1 Dataset — (ZIP) [file pone.0328537.s001.zip › S1 Dataset/Dataset1/CFA (Null model).tif]

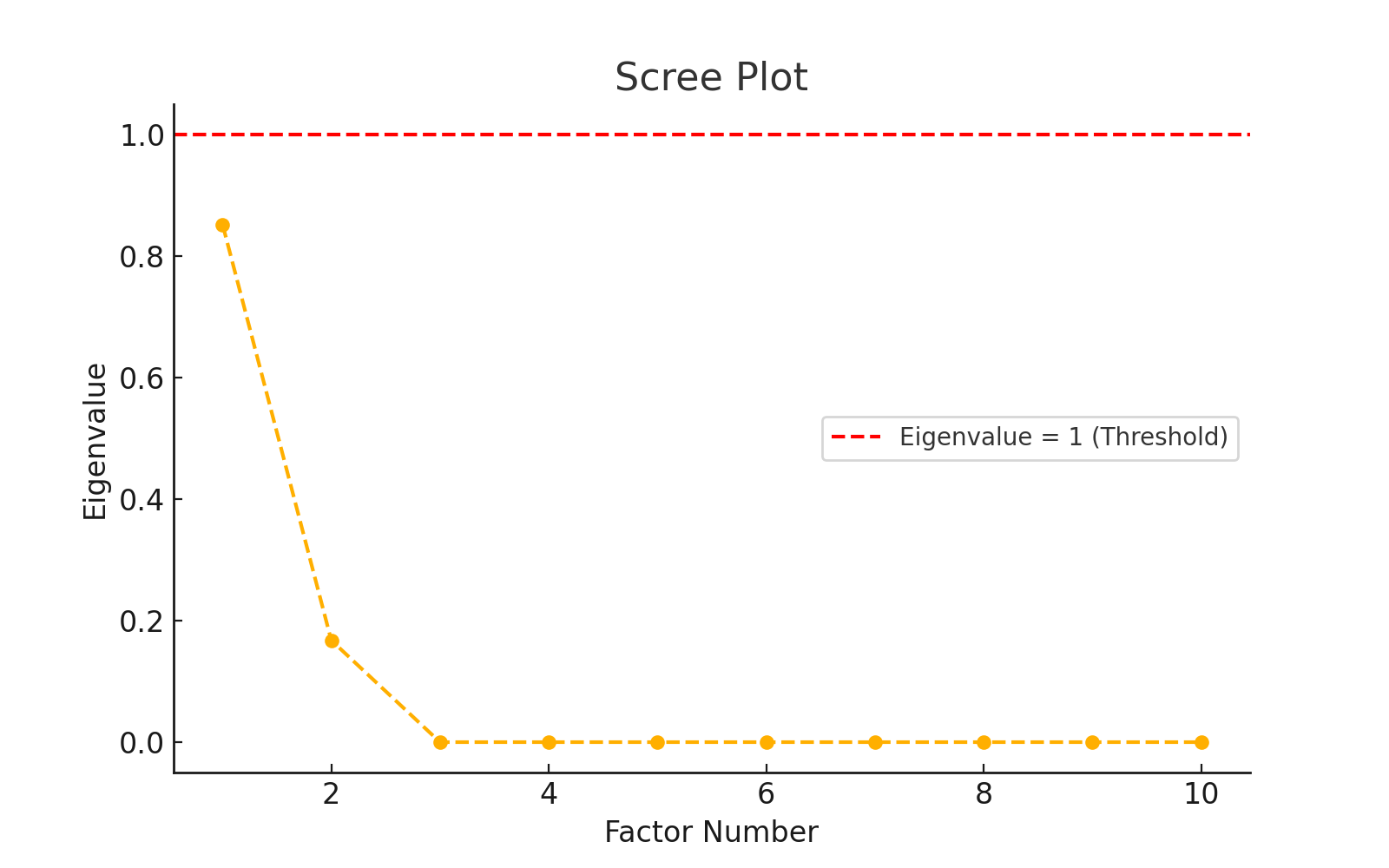

Supplement: S1 Dataset — (ZIP) [file pone.0328537.s001.zip › S1 Dataset/Dataset2/Scree_Plot.tif]
