# Supplementary material for: The efficacy of promoting sustained shared thinking through the use of activity books on parental empowerment; A quasi-experimental study
Source: PLoS One. 2025 Jul 18;20(7):e0328537. doi: 10.1371/journal.pone.0328537 (PMC12273987; doi:10.1371/journal.pone.0328537)
Supplement: S1 Protocol — (ZIP) [file pone.0328537.s004.zip › S4 Protocol/1-Certificate of Approval.pdf]

681 SAMSEN ROAD, DUSIT, BANGKOK 10300
Tel. 0-2244-3843

COA 091/2565

# INSTITUTIONAL REVIEW BOARD
# FACULTY OF MEDICINE VAJIRA HOSPITAL
# CERTIFICATE OF APPROVAL

**The Institutional Review Board of the Faculty of Medicine Vajira Hospital, is in full compliance with the International guidelines for human research protection as Declaration of Helsinki, The Belmont Report, CIOMS Guideline and International Conference on Harmonization in Good Clinical Practice (ICH-GCP)**

| | |
|---|---|
| PROTOCOL TITLE | The impact of sustained shared thinking based activity on parental empowerment<br>ประสิทธิผลของกิจกรรมที่เน้นการคิดร่วมกันอย่างต่อเนื่องในการเสริมพลังให้กับผู้ปกครอง |
| STUDY CODE | 289/64 FB |
| PRINCIPAL INVESTIGATOR | Asst. Prof. Kamolvisa Techapoonpon, MD |
| AFFILIATION | Department of Psychiatry Faculty of Medicine Vajira Hospital<br>Navamindradhiraj University |

APPROVAL DOCUMENTS

1. Research protocol **version 3 date 18 April 2022**
2. Informed consent documents **version 3 date 18 April 2022**
3. Case Record Form and questionnaire
4. Recruitment poster
5. Investigator's CV and ICH-GCP Training Certificate and declaration of conflict of interest
   - 5.1 Investigator's
     1) Asst. Prof. Kamolvisa Techapoonpon, MD
   - 5.2 Co-Investigator's
     1) May Sripattanaskul 2) Niyata Limpiti
     3) Kahwei Yoong

**Signature**..........................................................................

**(Professor Siriwan Tangjitgamol, MD)**
**Chairman**
**Vajira Institutional Review Board**

| | | | |
|---|---|---|---|
| <u>Initial Approval</u> | | <u>Number of Approval</u>: 1 | |
| Date of Approval | 25/04/2022 | Date of Approval | 04/09/2024 |
| Approval Expire Date | 24/04/2023 | Approval Expire Date | 24/04/2025 |

This protocol was reviewed in RM 14/64 agenda 4.1
Approval is granted subject to the following conditions: (see back of this Certificate)



สำนักงานคณะกรรมการพิจารณาจริยธรรมการวิจัย (ตึกเวชศาสตร์ฟื้นฟู ชั้น 5)
คณะแพทยศาสตร์วชิรพยาบาล มหาวิทยาลัยนวมินทราธิราช
681 ถนนสามเสน แขวงวชิรพยาบาล เขตดุสิต กรุงเทพ ฯ 10300
โทรศัพท์ 0-2244-3843

**All approved investigators must comply with the following conditions:**

1. Strictly conduct the research as required by the protocol;
2. Use only the information sheet, consent form (and recruitment materials, if any) veering the Institutional Review Board's seal of approval; and return one copy of such documents of the first subject recruited to the Institutional Review Board (IRB) for the record;
3. Report to the Institutional Review Board any serious adverse event, any Suspected Unexpected Serious Adverse Reaction: SUSAR which occur in Faculty of Medicine Vajira Hospital (Follow FERCIT AG Guidance, Published in June 2011)
4. Provide reports to the Institutional Review Board concerning the progress of the research upon the specified period of time or when requested; **(12 Months)**
5. If the study cannot be finished within the expire date of the approval certificate, the investigator is obliged to reapply for approval at least one month before the date of expiration.

สำนักงานคณะกรรมการพิจารณาจริยธรรมการวิจัย (ตึกเวชศาสตร์ฟื้นฟู ชั้น 5)
คณะแพทยศาสตร์วชิรพยาบาล มหาวิทยาลัยนวมินทราธิราช
681 ถนนสามเสน แขวงวชิรพยาบาล เขตดุสิต กรุงเทพ ฯ 10300
โทรศัพท์ 0-2244-3843
