# Supplementary material for: The efficacy of promoting sustained shared thinking through the use of activity books on parental empowerment; A quasi-experimental study
Source: PLoS One. 2025 Jul 18;20(7):e0328537. doi: 10.1371/journal.pone.0328537 (PMC12273987; doi:10.1371/journal.pone.0328537)
Supplement: S1 Protocol — (ZIP) [file pone.0328537.s004.zip › S4 Protocol/2-Approval of Amendment.pdf]

681 SAMSEN ROAD, DUSIT, BANGKOK 10300

Tel. 0-2244-3843

Amendment...1.../...2024...

INSTITUTIONAL REVIEW BOARD
FACULTY OF MEDICINE VAJIRA HOSPITAL
APPROVAL OF AMENDMENT

This document in a record of review and approve / acceptance of study protocol

Protocol Title The impact of sustained shared thinking based activity on parental empowerment
ประสิทธิผลของกิจกรรมที่เน้นการคิดร่วมกันอย่างต่อเนื่องในการเสริมพลังให้กับผู้ปกครอง

Principal Investigator Asst. Prof. Kamolvisa Techapoonpon, M.D.

Study Center Faculty of Medicine Vajira Hospital

Study Code 289/64 FB

Approval amendment issue

1. ขอเพิ่มชื่อหัวหน้าโครงการวิจัย ราย ผศ.นพ.วิศรัชต์ พฤฒิถาวร

Approval documents

1. Protocol **version 4 Date 23 August 2024**
2. Investigator's CV and ICH-GCP training certificate and declaration of conflict of interest
   1) Asst. Prof. Wisarat Pruttithavorn, M.D.

Date of review and Approval by reviewer: .....24...../.....09...../.....2024.....

Date of acknowledge in board meeting: .....11...../.....10...../.....2024..... (RM...10/67...)

This Institutional Review Board of the Faculty of Medicine Vajira Hospital is in full compliance with the International guidelines for human research protection such as Declaration of Helsinki, The Belmont Report, CIOMS Guideline and International Conference on Harmonization in Good Clinical Practice (ICH-GCP).

Signature...

(Professor Siriwan Tangjitgamol, MD)

Chairman

Vajira Institutional Review Board
