# Supplementary material for: The efficacy of promoting sustained shared thinking through the use of activity books on parental empowerment; A quasi-experimental study
Source: PLoS One. 2025 Jul 18;20(7):e0328537. doi: 10.1371/journal.pone.0328537 (PMC12273987; doi:10.1371/journal.pone.0328537)
Supplement: S1 Protocol — (ZIP) [file pone.0328537.s004.zip › S4 Protocol/3-Trial study Protocol from VIRB.pdf]

แบบเสนอโครงการวิจัย
คณะกรรมการพิจารณาจริยธรรมการวิจัย คณะแพทยศาสตร์วชิรพยาบาล

1. ชื่อโครงการ

ชื่อภาษาไทย ประสิทธิผลของกิจกรรมที่เน้นการคิดร่วมกันอย่างต่อเนื่องในการเสริมพลังให้กับผู้ปกครอง

ชื่อภาษาอังกฤษ The impact of sustained shared thinking based activity on parental empowerment

2. ชื่อหัวหน้าโครงการ

2.1 ชื่อภาษาไทย นางสาว xxxxxxxx
ชื่อภาษาอังกฤษ Miss Kxxxxxxxx
ตำแหน่ง ผู้ช่วยศาสตราจารย์
ภาควิชา จิตเวชศาสตร์
คณะแพทยศาสตร์วxxxxxxxx
โทรศัพท์ 0-2XXX-XXXX มือถือ 0XXXXXXXXX
E-mail: XXXXXXXXX
งานที่รับผิดชอบ ออกแบบงานวิจัย ยื่นขอจริยธรรม ขอทุน วิเคราะห์ข้อมูล สรุปผล วิจารณ์ผล ร่างต้นฉบับ

2.2 ชื่อภาษาไทย นาย วิxxxxxxxx
ชื่อภาษาอังกฤษ MR. Wxxxxxxxx
ตำแหน่ง ผู้ช่วยศาสตราจารย์
ภาควิชา จิตเวชศาสตร์
คณะแพทยศาสตร์ว xxxxxxxx
โทรศัพท์ 0- XXX-XXXX
E-mail: XXXXXXXXX
งานที่รับผิดชอบ สรุปผล วิจารณ์ผล ร่างต้นฉบับ ส่งตีพิมพ์

3. ชื่อผู้ร่วมวิจัย

3.1 ชื่อภาษาไทย นางสาว เมxxxxxxxx
ชื่อภาษาอังกฤษ Mxxxxxxxx
ตำแหน่ง ผู้ก่อตั้งบริษัทxxxxxxจำกัด ซึ่งเป็นบริษัทให้คำปรึกษาเพื่อพัฒนาองค์กร
ภาควิชา -
โทรศัพท์ XXXXXXXXX
E-mail: XXXXXXXXX
XXXXXXXXX

สำนักงานคณะกรรมการพิจารณาจริยธรรมการวิจัย (ตึกเวชศาสตร์ฟื้นฟู ชั้น 5)
คณะแพทยศาสตร์วชิรพยาบาล มหาวิทยาลัยนวมินทราธิราช
681 ถนนสามเสน แขวงวชิรพยาบาล เขตดุสิต กรุงเทพ ฯ 10300
โทรศัพท์ 0-2244-3843

	**งานที่รับผิดชอบ**	ออกแบบงานวิจัย ออกแบบกิจกรรมที่ใช้ทดลอง ร่างต้นฉบับ
**3.2**	**ชื่อภาษาไทย**	นางสาว นิXXXXXXXXX
	**ชื่อภาษาอังกฤษ**	NXXXXXXXXX
	**ตำแหน่ง**	ผู้ร่วมก่อตั้งบริษัท P XXXXXXXXX ซึ่งเป็นบริษัทให้คำปรึกษาในการออก-แบบการเรียนรู้
	**ภาควิชา**	-
	**โทรศัพท์**	XXXXXXXXX
	**E-mail:**	XXXXXXXXX
	**งานที่รับผิดชอบ**	ออกแบบงานวิจัย ออกแบบกิจกรรมที่ใช้ทดลอง ร่างต้นฉบับ
**3.3**	**ชื่อภาษาไทย**	นางสาว คXXXXXXXXX
	**ชื่อภาษาอังกฤษ**	KXXXXXXXXX
	**ตำแหน่ง**	ผู้ร่วมก่อตั้งบริษัท PXXXXXXXXX ซึ่งเป็นบริษัทให้คำปรึกษาในการออก-แบบการเรียนรู้
	**ภาควิชา**	-
	**โทรศัพท์**	-
	**E-mail:**	XXXXXXXXX
	**งานที่รับผิดชอบ**	ออกแบบงานวิจัย ออกแบบกิจกรรมที่ใช้ทดลอง ร่างต้นฉบับ

4. ชื่อแพทย์ผู้จะดูแล / ผู้รับผิดชอบผู้ถูกวิจัย

**4.1**	**ชื่อ**	นางสาว กXXXXXXXXX
	**ที่ทำงาน**	ภาควิชาจิตเวชศาสตร์ คณะแพทยศาสตร์ว XXXXXXXXX
	**โทรศัพท์**	XXXXXXXXX มือถือ XXXXXXXXX
	**งานที่รับผิดชอบ**	ออกแบบงานวิจัย ยื่นขอจริยธรรม ขอทุน วิเคราะห์ข้อมูล สรุปผล วิจารณ์ผล ร่างต้นฉบับ

5. ความสำคัญและที่มาของปัญหา

ช่วงเวลาปฐมวัย (early childhood) หรือวัยตั้งแต่แรกเกิดไปจนถึงถึงอายุไม่เกิน 6 ปี ถือเป็นวัยมีความสำคัญอย่างยิ่งในการพัฒนาทาง

สำนักงานคณะกรรมการพิจารณาจริยธรรมการวิจัย (ตึกเวชศาสตร์ฟื้นฟู ชั้น 5)
คณะแพทยศาสตร์วชิรพยาบาล มหาวิทยาลัยนวมินทราธิราช
681 ถนนสามเสน แขวงวชิรพยาบาล เขตดุสิต กรุงเทพ ฯ 10300
โทรศัพท์ 0-2244-3843

สติปัญญาและอารมณ์ของมนุษย์ เนื่องจากเป็นช่วงที่สมองพัฒนาอย่างรวดเร็ว และเมื่อผ่านช่วงนี้ไปแล้วไปแล้ว การย้อนกลับไปแก้ไขข้อผิดพลาดทำได้ยาก[1] ซึ่งการพัฒนาดังกล่าวขึ้นกับการกระตุ้นทางสังคมและสิ่งแวดล้อมเป็นสำคัญ ดังนั้นครอบครัวจึงมีบทบาทสำคัญอย่างยิ่ง เพราะเป็นช่วงวัยที่เด็กใช้เวลาอยู่กับครอบครัวมากที่สุด[2] จากการศึกษาพบว่าการที่ครอบครัวให้เวลาดูแลบุตรหลานอย่างมีคุณภาพนั้นสามารถช่วยส่งเสริมผลการเรียนและลดปัญหาพฤติกรรมของเด็กในอนาคตได้อย่างมีนัยสำคัญ นอกจากการเสริมพัฒนาการในช่วงนี้สัมพันธ์กับการมีรายได้ที่เพิ่มขึ้นเมื่อเด็กเข้าสู่วัยผู้ใหญ่ได้ถึงร้อยละ 25[3]

อย่างไรก็ตามในปัจจุบันปฐมวัยกลับไม่ได้รับการกระตุ้นจากครอบครัวมากเท่าที่ควรและกำลังสูญเสียโอกาสที่จะพัฒนาศักยภาพได้อย่างเต็มที่ จากการสำรวจพบว่าร้อยละ 43 ของเด็กปฐมวัยในกลุ่มประเทศรายได้น้อยถึงรายได้ปานกลางกำลังประสบปัญหาดังกล่าว[4] ซึ่งปัญหานี้เกิดจากหลายสาเหตุผนวกกัน อาทิ การขาดความรู้และทัศนคติที่เหมาะสมต่อการเลี้ยงบุตรหลานในช่วงปฐมวัย[5] การที่มารดาในยุคปัจจุบันมีอัตราการทำงานนอกบ้านมากขึ้นเมื่อเทียบกับในอดีตทำให้ไม่สามารถดูแลลูกได้เต็มที่[6] การมีความเชื่อว่าการเรียนเป็นหน้าที่ของโรงเรียนไม่ใช่ผู้ปกครอง หรือแม้กระทั่งการมีความคิดว่าตัวเองไม่มีศักยภาพเพียงพอในการส่งเสริมพัฒนาการของลูก[7] นอกจากนี้ผู้ปกครองของเด็กจำนวนไม่น้อยให้บุตรหลานใช้เวลาส่วนใหญ่ไปกับโทรศัพท์มือถือเป็นเหตุให้อัตราการติดโทรศัพท์มือถือในเด็กปฐมวัยมีมากขึ้น[8] ดังนั้นการส่งเสริมให้ผู้ปกครองใช้เวลากับเด็กปฐมวัยอย่างมีประสิทธิภาพจึงเป็นเรื่องที่สำคัญอย่างยิ่ง

หนึ่งในปัจจัยสำคัญที่มีส่วนกำหนดบทบาทของผู้ปกครองในการส่งเสริมพัฒนาการของลูก ได้แก่ การเสริมพลังในผู้ปกครอง หรือ parental empowerment ซึ่งหมายถึง กระบวนการรวมไปถึงผลลัพธ์ภายในจิตใจของผู้ปกครองว่าตนเองสามารถควบคุม จัดการ และรับมือกับบทบาทการเป็นผู้ปกครองได้อย่างดี[9] จากงานวิจัยในอดีต พบว่า ผู้ปกครองที่รู้สึกมีพลังในบทบาทผู้ปกครอง จะทำหน้าที่ผู้ปกครองได้อย่างดี รวมทั้งการเสริมพลังในผู้ปกครองยังสัมพันธ์กับมีส่วนร่วมในการส่งเสริมพัฒนาการและการเรียนของเด็กที่โรงเรียนอีกด้วย[10] ด้วยเหตุนี้การเพิ่มการเสริมพลังในผู้ปกครองจึงเป็นตัวกลางในการเสริมพัฒนาการให้กับเด็กที่มีความยั่งยืน

ทางทีมผู้วิจัยจึงเล็งเห็นประเด็นดังกล่าวและมีเป้าหมายที่จะสร้างกิจกรรมที่สามารถช่วยเพิ่มการเสริมพลังให้กับผู้ปกครองเพื่อนำไปสู่การส่งเสริมพัฒนาการด้านความคิดและการเรียนของลูกต่อไป โดยจะเน้นเฉพาะกลุ่มผู้ปกครองที่ต้องทำงานประจำซึ่งมีเวลาจำกัดในการเสริมพัฒนาการให้กับลูก โดยอาศัยหลักการในการเสริมพลัง คือ การให้ผู้ปกครองได้

เป็นตัวกลางของการเปลี่ยนแปลงของลูก (Parents as Agents of Change)[11] กล่าวคือ เป็นกิจกรรมที่เสริมพัฒนาการด้านสติปัญญาให้กับเด็กโดยมีผู้ปกครองเป็นสื่อกลาง ดังนั้นกิจกรรมดังกล่าวจะอาศัยการมีปฏิสัมพันธ์ระหว่างเด็กกับผู้ปกครองเป็นกลไกหลัก จากการค้นคว้าการศึกษาและงานวิจัยในอดีต กิจกรรมที่เน้นการคิดร่วมกันอย่างต่อเนื่อง (sustained shared thinking) สามารถตอบโจทย์งานวิจัยชิ้นนี้ได้

Sustained Shared Thinking (SST) คือ การที่บุคคลตั้งแต่ 2 คนขึ้นไประดมสมองแลกเปลี่ยนความคิดเพื่อให้เกิดผลผลิตทางความคิด เช่น การแก้ปัญหา การประมวลผล และการบรรยาย เป็นต้น[12] จากการศึกษาในอดีตพบว่า SST เป็นรูปแบบการกระตุ้นพัฒนาการด้านสติปัญญาที่ได้ผลดีในเด็กก่อนวัยเรียน[13] การบรรจุ SST ลงในการเล่นทำให้เด็กสนุกและอยากแบ่งปันความคิดได้นานขึ้น[14] ดังนั้นทางทีมผู้วิจัยจึงบรรจุ SST ลงในกิจกรรมการเล่นที่ผู้ปกครองสามารถทำกับบุตรหลานได้ โดยใช้คู่มือที่มีรูปแบบน่าสนใจ (workbook)เป็นสื่อกลาง เนื่องจากสะดวกและทำตามได้ง่ายสำหรับผู้ปกครอง กิจกรรมในคู่มือจะสอดคล้องกับระดับพัฒนาการของเด็กก่อนวัยเรียน

สำหรับในส่วนของการประเมิน parental empowerment นั้น ทีมผู้วิจัยได้พัฒนาแบบประเมินขึ้นโดยอ้างอิงมาจากทฤษฎีของ Zimmerman[15] ซึ่งแบ่ง empowerment ออกเป็น 3 องค์ประกอบ ได้แก่ องค์ประกอบระดับภายในจิตใจ ระดับปฏิสัมพันธ์ และระดับพฤติกรรม และใน 3 องค์ประกอบดังกล่าวจะแบ่งออกเป็น 12 หัวข้อย่อย ซึ่งแบบสอบถามที่ทางทีมผู้วิจัยพัฒนาขึ้นนั้นครอบคลุมทั้ง 12 หัวข้อดังกล่าว

ในปัจจุบันยังไม่มีงานวิจัยใดที่ศึกษาถึงประสิทธิผลของกิจกรรมที่เน้นการคิดร่วมกันอย่างต่อเนื่องในการเสริมพลังให้กับผู้ปกครองที่ทำงานประจำ ดังนั้น งานวิจัยฉบับนี้จึงมีวัตถุประสงค์หลักเพื่อศึกษาประสิทธิผลของกิจกรรมที่เน้นการคิดร่วมกันอย่างต่อเนื่องในการเสริมพลังให้กับผู้ปกครอง นอกจากนี้ยังมีวัตถุประสงค์รองเพื่อศึกษาตัวกลางหรือกลไกที่เชื่อมระหว่างการคิดร่วมกันอย่างต่อเนื่องและการเสริมพลังในผู้ปกครอง ซึ่งในที่นี้คือ การรับรู้ถึงความก้าวหน้าของพัฒนาการของลูกที่เกิดจากผู้ปกครองเป็นผู้กระตุ้น โดยคำตอบที่ได้จากงานวิจัยชิ้นนี้จะสามารถใช้เป็นแนวทางในการส่งเสริมพัฒนาการของเด็กอย่างยั่งยืนต่อไปในอนาคต

6. วัตถุประสงค์

6.1 วัตถุประสงค์หลัก

-เพื่อศึกษาประสิทธิผลของกิจกรรมที่เน้นการคิดร่วมกันอย่างต่อเนื่องในการเสริมพลังให้กับผู้ปกครอง

6.2 วัตถุประสงค์รอง

สำนักงานคณะกรรมการพิจารณาจริยธรรมการวิจัย (ตึกเวชศาสตร์ฟื้นฟู ชั้น 5)
คณะแพทยศาสตร์วชิรพยาบาล มหาวิทยาลัยนวมินทราธิราช
681 ถนนสามเสน แขวงวชิรพยาบาล เขตดุสิต กรุงเทพ ฯ 10300
โทรศัพท์ 0-2244-3843

-เพื่อศึกษาประสิทธิผลของกิจกรรมที่เน้นการแบ่งปันความคิดกับการรับรู้ถึงความก้าวหน้าของพัฒนาการของลูกที่เกิดจากผู้ปกครองเป็นผู้กระตุ้น

-เพื่อศึกษาปัจจัยที่มีผลต่อประสิทธิผลของกิจกรรมที่เน้นการคิดร่วมกันอย่างต่อเนื่องในการเสริมพลังให้กับผู้ปกครองและการรับรู้ถึงความก้าวหน้าของพัฒนาการของลูก

7. กรอบแนวคิดการวิจัย

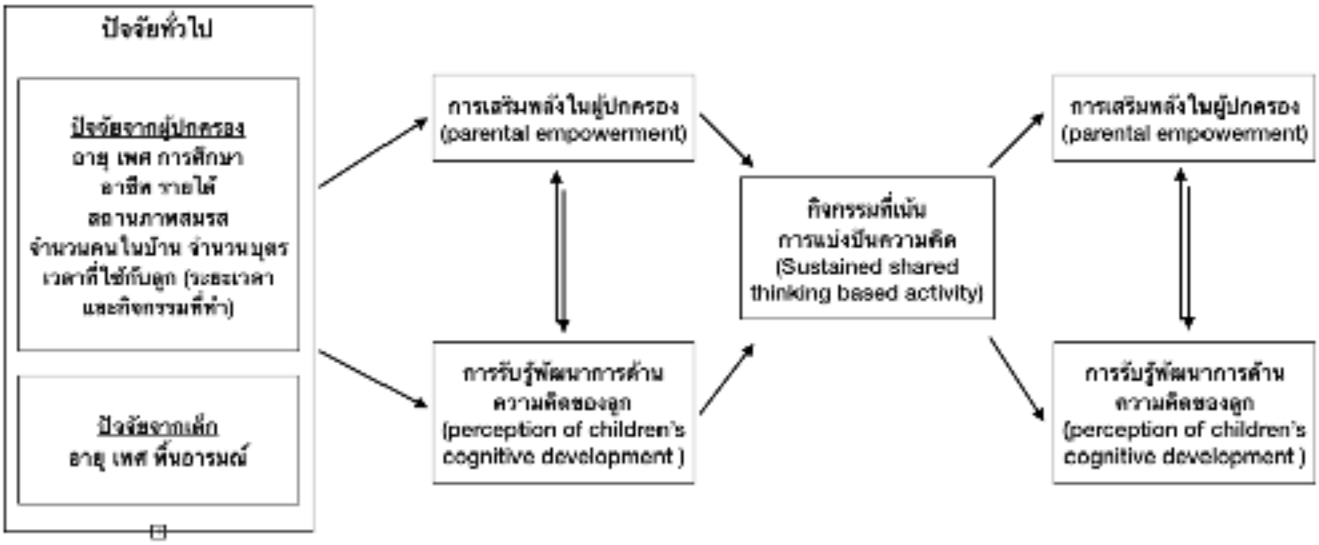

8. รูปแบบการวิจัยและระเบียบวิธีวิจัย

8.1 รูปแบบการวิจัย

งานวิจัยกึ่งทดลอง (Quasi-experimental study)

8.2 กลุ่มประชากรเป้าหมาย

- บิดาหรือมารดาและลูกที่มีอายุระหว่าง 4-6 ปี

8.3 เกณฑ์การคัดเข้า เกณฑ์การคัดออก และเกณฑ์การหยุดการวิจัย

เกณฑ์การคัดเข้า (Inclusion Criteria)

- เป็นบิดาหรือมารดาที่มีอายุมากกว่า 18 ปี
- เป็นบิดาหรือมารดาที่มีลูกอายุระหว่าง 4-6 ปี

สำนักงานคณะกรรมการพิจารณาจริยธรรมการวิจัย (ตึกเวชศาสตร์ฟื้นฟู ชั้น 5) DATE:23 สิงหาคม 2567
คณะแพทยศาสตร์วชิรพยาบาล มหาวิทยาลัยนวมินทราธิราช
681 ถนนสามเสน แขวงวชิรพยาบาล เขตดุสิต กรุงเทพ ฯ 10300
โทรศัพท์ 0-2244-3843

- เป็นบิดาหรือมารดาทำงานประจำ
- เป็นบิดาหรือมารดาที่รู้สึกขาดพลังในการเลี้ยงลูก (ตอบแบบสอบถามในขั้นตอนรวบรวมอาสาสมัคร ว่ามีพลังในการเลี้ยงลูกอยู่ในเกณฑ์น้อย, ค่อนข้างน้อย จากเกณฑ์ทั้งหมด 5 ตัวเลือก ได้แก่ น้อย,ค่อนข้างน้อย, ปานกลาง, ค่อนข้างมาก, มาก)

เกณฑ์การคัดออก (Exclusion Criteria)

- บิดาหรือมารดามีโรคประจำตัวทางจิตเวช
- ลูกมีปัญหาพัฒนาการ (Neurodevelopmental disorders)
- ลูกมีปัญหาโรคทางกายที่มียาทานประจำ หรือ ต้องพบแพทย์ประจำ

เกณฑ์การหยุดการวิจัย (Termination criteria)

- เมื่อผู้เข้าร่วมวิจัยต้องการออกจากโครงการ

8.4 จำนวนประชากร

ขนาดตัวอย่าง (Sample size)

การศึกษาแบบกึ่งทดลอง มีวัตถุประสงค์เพื่อเปรียบเทียบค่าเฉลี่ยของก่อนและหลังการทดลอง และเนื่องจากไม่มีการศึกษามาก่อนหน้าจึงไม่มีค่าสถิติในการอ้างอิงเพื่อใช้ในการคำนวณขนาดตัวอย่างตามวิธีการ ประมาณค่าขนาดตัวอย่างโดยใช้สูตรคำนวณขนาดตัวอย่าง ดังนั้นการกำหนดขนาดตัวอย่างในการศึกษานี้ใช้การ ประมาณค่าขนาดตัวอย่างจากโปรแกรม G power version 3.1.9.7 โดยกำหนด statistical test ชนิด paired t tests และ power analysis ชนิด A priori: Compute required sample size (ระดับนัยสำคัญ $\alpha = 0.05$ อำนาจการทดสอบ 0.9(90%) และผู้วิจัยกำหนดค่า Effect size d เท่ากับ 0.5 (Medium)[16] เนื่องจากไม่มีการศึกษาอ้างอิงค่าสถิติที่ใช้ในการคำนวณค่า Effect size ทั้งนี้ขนาดตัวอย่างที่คำนวณได้จากโปรแกรมได้จำนวน) ตัวอย่างที่จะต้องใช้ทั้งหมดไม่นอยกว่า 44 คน และได้ปรับเพิ่มขนาดตัวอย่างเนื่องจากป้องกันการสูญหายของตัวอย่างจากการติดตามตาม/การมีข้อมูลขาดหาย/การตอบข้อมูลไม่ครบถ้วนสมบูรณ์กรณีเก็บข้อมูลโดยใช้แบบสอบถาม ร้อยละ 20 ขนาดตัวอย่างที่คำนวณจากสูตร[17] ดังนั้นการศึกษานี้จึงใช้กลุ่มตัวอย่างทั้งหมด 55 คน

8.5 ตัวแปร

ตัวแปรอิสระ

สำนักงานคณะกรรมการพิจารณาจริยธรรมการวิจัย (ตึกเวชศาสตร์ฟื้นฟู ชั้น 5)
คณะแพทยศาสตร์วชิรพยาบาล มหาวิทยาลัยนวมินทราธิราช
681 ถนนสามเสน แขวงวชิรพยาบาล เขตดุสิต กรุงเทพ ฯ 10300
โทรศัพท์ 0-2244-3843

- ปัจจัยจากผู้ปกครอง - อายุ เพศ การศึกษา อาชีพ รายได้ สถานภาพสมรส จำนวนคนในบ้าน จำนวนบุตร ระยะเวลาที่ใช้กับลูก
- ปัจจัยจากเด็ก - อายุ เพศ พื้นอารมณ์
- กิจกรรมที่เน้นการคิดร่วมกันอย่างต่อเนื่อง (Sustained shared thinking based activity)

ตัวแปรตาม

- การเสริมพลังในผู้ปกครอง (parental empowerment)
- การรับรู้พัฒนาการด้านความคิดของลูก(perception of children’s development)

8.6 นิยามตัวแปร

- กิจกรรมที่เน้นการการคิดร่วมกันอย่างต่อเนื่อง (Sustained shared thinking based activity) คือ กิจกรรมที่ทำระหว่าง ผู้ปกครองและเด็กซึ่งประกอบด้วยระดมสมองแลกเปลี่ยนความคิด เพื่อให้เกิดผลผลิตทางความคิด โดยมีเครื่องมือที่ทีมผู้วิจัยผลิตขึ้น เป็นสื่อกลาง
- การเสริมพลังในผู้ปกครอง (parental empowerment) คือ กระบวนการรวมไปถึงผลลัพธ์ภายในจิตใจของผู้ปกครองว่าตนเอง สามารถควบคุม จัดการ และรับมือกับบทบทการเป็นผู้ปกครองได้ อย่างดี
- การรับรู้การรับรู้ถึงความก้าวหน้าของพัฒนาการของลูก คือ การรับรู้ของผู้ปกครองที่มีต่อการเพิ่มขึ้นของความสามารถใน การคิด การให้เหตุผลของลูก และด้านภาษา

8.7 กระบวนการขอคำยินยอมแก่อาสาสมัครให้เข้าร่วมการวิจัยและการได้มาซึ่งอาสาสมัคร (inform consent process and recruitment process)

โครงการวิจัยจะได้รับการประชาสัมพันธ์ผ่านช่องทางสื่อสังคมออนไลน์ ได้แก่ Facebook, Instagram, Twitter และ ส่งต่อทาง Line application และประชาสัมพันธ์ในโรงเรียนที่มีนักเรียนชั้นอนุบาล

ผู้ที่สนใจเข้าร่วมจะสามารถติดต่อทีมผู้วิจัยจาก QR code ที่ปรากฏในโปสเตอร์ประชาสัมพันธ์ที่เผยแพร่ทางสื่อออนไลน์ จากนั้นผู้ที่สนใจสามารถ scan QR code และกรอกข้อมูลเบื้องต้นตามเกณ์การคัดเข้าและเกณฑ์การคัดออก ถ้าหากมีคุณสมบัติเข้าตามเกณฑ์ ทางทีมผู้วิจัยจะติดต่อผู้สนใจต่อไป

หลังจากนั้นผู้วิจัยจะเป็นผู้ให้ข้อมูลการวิจัยและขอความยินยอมจากผู้เข้าร่วมโครงการวิจัยโดยตรง โดยให้เอกสารข้อมูลเกี่ยวกับการวิจัย และเอกสารขอความยินยอมเป็นผู้เข้าร่วมวิจัย จากนั้นจะมีการยืนยันว่าอาสาสมัครมีตัวตนโดยการถ่ายรูปร่วมกับลูกและส่งให้กับทีมผู้วิจัย

8.8 วิธีดำเนินการวิจัย (Methodology)

8.8.1 ขออนุญาตดำเนินการวิจัยจากคณะกรรมการพิจารณาจริยธรรมการวิจัยคณะแพทยศาสตร์วชิรพยาบาล มหาวิทยาลัยนวมินทราธิราช

8.8.2 ทีมผู้วิจัยพัฒนาแบบสอบถามการเสริมพลังในผู้ปกครอง (parental empowerment) และแบบสอบถาม การรับรู้พัฒนาการด้านความคิดของลูก(perception of children's cognitive development)

8.8.3 การทบทวนโดยผู้ทรงคุณวุฒิ (Review)

8.8.4 การหาความเที่ยง (Reliability) และ ความตรง (Validity)

8.8.5 พัฒนากิจกรรมที่เน้นการแบ่งปันความคิดที่จะใช้ในโครงการวิจัย

8.8.6 ประชาสัมพันธ์โครงการวิจัยผ่านชองทางสื่อออนไลน์ (Facebook, Instagram, Twitter และ ส่งต่อทาง Line application) และติดต่อโรงเรียนที่มีการเรียนการสอนระดับชั้นอนุบาลเพื่อเป็นการเข้าถึงผู้ปกครองอีกหนึ่งช่องทาง

8.8.7 คัดเลือกกลุ่มตัวอย่างผู้ป่วยตามเกณฑ์การคัดเข้า (inclusion criteria) และเกณฑ์การคัดออก (exclusion criteria)

8.8.8ให้ข้อมูลเกี่ยวกับการวิจัย และขอความยินยอม ผ่าน application Line (การขอความยินยอมทำโดยการกรอกชื่อ-สกุล และกดปุ่มยินยอมซึ่งจะปรากฏท้ายใบขอความยินยอมซึ่งอยู่ในรูปแบบ google form) นอกจากนี้มีการยืนยันว่าอาสาสมัครมีตัวตนโดยการถ่ายรูปร่วมกับลูกและส่งให้กับทีมผู้วิจัย

8.8.9 ดำเนินการเก็บข้อมูลวิจัยครั้งแรก ได้แก่ ข้อมูลทั่วไป แบบสอบถามการเสริมพลังในผู้ปกครอง (parental empowerment) และแบบสอบถามการรับรู้พัฒนาการของลูก(Cognitive and literacy skills)โดยใช้เวลาตอบแบบสอบถามก่อนทำกิจกรรมประมาณ 15 นาที ใช้วิธีการตอบคำถามใน Google form ผ่านทาง Application Line และอาจสัมภาษณ์ทางโทรศัพท์ในกรณีที่ติดต่ออาสาสมัครทางโทรศัพท์ หรือผู้ป่วยไม่สะดวกตอบคำถามทาง Google form

8.8.10 ดำเนินการอธิบายรายละเอียดการทำกิจกรรมกับลูกอย่างละเอียด ซึ่งผู้ปกครองจะต้องทำกิจกรรมกับลูกเป็นเวลา 6 สัปดาห์ โดยมีคู่มือและเครื่องมือที่ออกแบบและจัดทำโดยทีมผู้วิจัย หนังสือกิจกรรมจะถูกจัดส่งไปตามที่อยู่ที่อาสาสมัครให้ไว้สัปดาห์ละครั้ง ให้ทำกิจกรรมสัปดาห์ละอย่างน้อย 1 ครั้ง แต่ละครั้งใช้เวลาประมาณ 25-30 นาที ภายหลังจากที่ผู้ปกครองทำกิจกรรมในแต่ละสัปดาห์เสร็จ ผู้ปกครองจะต้องถ่ายรูปผลงาน และ reflection ซึ่งจะเป็นคำถามที่อยู่หน้าสุดท้ายของแบบฝึกหัดส่งมาให้กับทีมผู้วิจัยทุกสัปดาห์

8.8.11 ดำเนินการเก็บข้อมูลหลังจากทำกิจกรรมครั้งที่ 3 และ 6 (ครั้งสุดท้าย) โดยตอบแบบสอบถามการเสริมพลังในผู้ปกครอง (parental empowerment) แบบสอบถามการรับรู้พัฒนาการของลูก (Cognitive and literacy skills) และคำถามปลายเปิดเกี่ยวกับประสบการณ์การทำกิจกรรม ใช้เวลาตอบแบบสอบถามประมาณ 15-20 นาที

8.8.12 ตรวจสอบความสมบูรณ์ของแบบสอบถามและนำข้อมูลที่ได้มาวิเคราะห์ทางสถิติ

8.9 เครื่องมือวัดตัวแปร

8.9.1 แบบสอบถามข้อมูลของผู้เข้าร่วมโครงการวิจัย ประกอบด้วยข้อมูลทั่วไปของผู้ปกครอง (ได้แก่ อายุ เพศ การศึกษา อาชีพ รายได้ สถานภาพสมรส ผู้ที่พักอาศัยด้วย จำนวนบุตร เวลาที่ใช้กับลูก (ระยะเวลาและกิจกรรมที่ทำ) และข้อมูลทั่วไปของเด็ก (ได้แก่ อายุ เพศ พื้นอารมณ์)

8.9.2 แบบประเมินการเสริมพลังในผู้ปกครอง (Parental empowerment)

8.9.3 แบบสอบถามการรับรู้พัฒนาการของลูก(perception of children’s development in cognitive and literacy skills)

8.9.4 แบบสอบถามเกี่ยวกับประสบการณ์ที่ได้รับหลังจากทำกิจกรรมในโครงการวิจัย

8.9.5 กิจกรรมที่เน้นการคิดร่วมกันอย่างต่อเนื่อง (sustained shared thinking based activity) มีรายละเอียดดังเอกสารแนบ

8.10 การเก็บรวบรวมข้อมูล

ดำเนินการเก็บข้อมูลโดยให้ผู้ที่เข้าเกณฑ์ตอบแบบสอบถามและแบบประเมิน โดยแยกบันทึกข้อมูลเป็นรายบุคคล ซึ่งจะไม่ปรากฏชื่อหรือข้อมูลที่สามารถระบุถึงตัวผู้เข้าร่วมโครงการวิจัย และจะมีเพียงทีมผู้วิจัยเท่านั้นที่สามารถเข้าถึงชุดข้อมูลนี้ได้ โดยทีมผู้ออกแบบแบบฝึกหัดจะไม่มีส่วนเกี่ยวข้องในการคัดเลือกผู้เข้าร่วมโครงการวิจัย

ตรวจสอบความครบถ้วนของข้อมูลและนำข้อมูลที่ได้มาตรวจให้คะแนนตามหลักเกณฑ์ เพื่อนำวิเคราะห์ข้อมูลทางสถิติต่อไป

8.11 การวิเคราะห์ข้อมูล

- สถิติเชิงพรรณนา โดยใช้สถิติพื้นฐาน ได้แก่ ร้อยละ (percentage) ค่าเฉลี่ย (mean) ส่วนเบี่ยงเบนมาตรฐาน (standard deviation)
- การเปรียบเทียบคะแนนการเสริมพลังในผู้ปกครอง (Parental empowerment) และคะแนนการรับรู้พัฒนาการด้าน

ความคิดของลูก(perception of children’s cognitive development) ก่อนและหลังทำกิจกรรมครั้งที่ 3 และ 6 โดยใช้ค่าสถิติ t (t-test)
- การทดสอบความสัมพันธ์ทางสถิติ โดยใช้สูตรสหสัมพันธ์ของเพียร์สัน (Pearson’s product moment correlation coefficient)
- การวิเคราะห์เชิงคุณภาพ (Qualitative analysis)
-โดยทีมผู้ออกแบบแบบฝึกหัดจะไม่มีส่วนเกี่ยวข้อง ในการวิเคราะห์ข้อมูล

8.12 ตารางการนำเสนอ (Dummy table)

ข้อมูลพื้นฐานของผู้ปกครอง	ความถี่ (n)	ร้อยละ (percentage)
อายุ		
เพศ		
การศึกษา		
อาชีพ		
รายได้		
สถานภาพสมรส		
จำนวนคนในบ้าน		
จำนวนบุตร		
ระยะเวลาที่ใช้กับลูก		
รูปแบบกิจกรรมที่ทำกับลูกเป็นประจำ		

ข้อมูลพื้นฐานของเด็ก	ความถี่ (n)	ร้อยละ (percentage)
อายุ		
เพศ		
พื้นอารมณ์		

แบบประเมินก่อนทำกิจกรรม	คะแนน	ค่าเบี่ยงเบนมาตรฐาน	ระดับ
parental empowerment			
cognitive development			
literacy skill			

สำนักงานคณะกรรมการพิจารณาจริยธรรมการวิจัย (ตึกเวชศาสตร์ฟื้นฟู ชั้น 5)
คณะแพทยศาสตร์วชิรพยาบาล มหาวิทยาลัยนวมินทราธิราช
681 ถนนสามเสน แขวงวชิรพยาบาล เขตดุสิต กรุงเทพ ฯ 10300
โทรศัพท์ 0-2244-3843

แบบประเมินหลังทำกิจกรรม	คะแนน		ค่าเบี่ยงเบนมาตรฐาน	
	หลังทำกิจกรรม ครั้งที่ 3 (คะแนน/ระดับ)	หลังทำกิจกรรม ครั้งที่ 6 (คะแนน/ระดับ)	หลังทำกิจกรรม ครั้งที่ 3 (คะแนน/ระดับ)	หลังทำกิจกรรม ครั้งที่ 6 (คะแนน/ระดับ)
parental empowerment				
cognitive development				
literacy skill				

ตัวแปร	ก่อนทำกิจกรรม (SD)	หลังทำกิจกรรมครั้งที่ 6 (SD)	t	p-value
parental empowerment				
cognitive development				
literacy skill				

9. ความเสี่ยงและผลข้างเคียงที่อาจเกิด

ความเสี่ยงทางด้านจิตใจ เนื่องจากการทำกิจกรรมกับลูกต้องอาศัยแรงกายแรงใจ และการตอบข้อคำถามบางข้ออาจส่งผลกระทบในแง่ลบต่อสุขภาพจิตได้ ดังนั้น ผู้ตอบแบบสอบถามมีสิทธิ์ที่จะไม่ตอบหรือยุติการตอบแบบสอบถามได้ รวมไปถึงสามารถถอนตัวจากการเข้าร่วมวิจัยได้ และถ้าหากผลกระทบต่อจิตใจรุนแรงทีมผู้วิจัยจะแนะนำให้เข้าสู่กระบวนการช่วยเหลือโดยนักจิตวิทยาหรือจิตแพทย์ คณะแพทยศาสตร์วชิรพยาบาล

10. ประโยชน์และผลกระทบที่คาดว่าจะได้รับ

- ประสิทธิผลของกิจกรรมที่เน้นการคิดร่วมกันอย่างต่อเนื่องในการเสริมพลังให้กับผู้ปกครอง
- ได้แบบสอบถามเพื่อวัดการเสริมพลังในผู้ปกครอง(Parental empowerment)

11. ข้อพิจารณาด้านจริยธรรม

การดำเนินงานวิจัยเป็นไปตามหลักจริยธรรมพื้นฐานของ Belmont Report ได้แก่

1)หลักการเคารพในตัวบุคคล ด้วยการให้ข้อมูลของโครงการวิจัยโดยไม่มีการปิดบังอาสาสมัคร และอาสาสมัครทุกท่านยินยอมเข้าร่วมงานวิจัยด้วยความสมัครใจ เอกสารที่เกี่ยวข้องกับงานวิจัยจะถูกเก็บรักษาและเข้าถึงได้เฉพาะผู้เกี่ยวข้องในโครงการวิจัย นำเสนอข้อมูลด้วยภาพรวม ไม่มีการระบุชื่อจริงของอาสา

สมัคร ก่อนดำเนินโครงการวิจัยจะต้องผ่านการพิจารณาอนุมัติจากคณะกรรมการพิจารณาจริยธรรมการวิจัย และอาสาสมัครหรือผู้แทนโดยชอบธรรมตามกฎหมาย จะต้องลงนามแสดงความยินยอมทุกคน

2) หลักผลประโยชน์ อาสาสมัครจะได้รับทราบความเสี่ยงและประโยชน์ที่เกิดขึ้นจากการเข้าร่วมโครงการวิจัย ทั้งนี้อาสาสมัครจะได้รับคำแนะนำการปฏิบัติตัวเมื่อพบว่าเป็นผู้ที่มีความผิดปกติอย่างใดอย่างหนึ่ง

3) หลักยุติธรรม โดยแม้ว่าไม่ต้องการเข้าร่วมโครงการวิจัยนี้ อาสาสมัครทุกคนจะได้รับการดูแลรักษาเต็มที่ตามมาตรฐานการรักษา

12. ข้อจำกัดของการวิจัย

การเข้าถึงผู้เข้าร่วมโครงการวิจัยจะทำโดยการติดต่อระยะไกล (distant contact) เท่านั้น โดยเฉพาะผ่าน application Line ดังนั้นอาจมีข้อจำกัดในรายที่ไม่สันทัดการใช้เทคโนโลยีชนิดนี้

13. อุปสรรคที่อาจเกิดขึ้นและแนวทางแก้ไข

โครงการวิจัยนี้ทำที่บ้านโดยผู้ปกครองเอง ต้องอาศัยความร่วมมือและความซื่อสัตย์ของผู้เข้าร่วมวิจัยเป็นสำคัญเนื่องจากต้องอาศัยเวลาและความพยายามพอสมควร ดังนั้นอาจมีอุปสรรคในแง่ของความร่วมมือได้ ทางทีมผู้วิจัยมีแนวทางการแก้ไขโดยการติดต่อสอบถาม ให้คำแนะนำเป็นระยะๆ และเปิดช่องทางให้ผู้เข้าร่วมวิจัยติดต่อทีมผู้วิจัยได้เสมอ นอกจากนี้ท้ายสุดของเล่มกิจกรรมจะมีคำถามสะท้อนความรู้สึก (reflection) ให้ผู้ปกครองกรอก ซึ่งผู้ปกครองต้องถ่ายภาพส่งทีมผู้วิจัยหลังทำกิจกรรมสำเร็จในทุกสัปดาห์

14. การบริหารงานวิจัยและตารางเวลาในการศึกษา

ลำดับ	กิจกรรม	เดือนที่												ผลผลิตส่งมอบ
		1	2	3	4	5	6	7	8	9	10	11	12	
1	ศึกษาปัญหาและกำหนดประเด็นการวิจัย	●												
2	ทบทวนวรรณกรรม และพัฒนาโครงร่างการวิจัย	●												
3	ขอพิจารณาจริยธรรมการวิจัยในมนุษย์		●	●										
4	พัฒนาและตรวจสอบคุณภาพเครื่องมือวิจัย				●									ได้แบบสอบถามเพื่อวัดการเสริมพลังในผู้ปกครองและกิจกรรมที่เน้นการแบ่งปันความคิด
5	ประสานงานและวางแผนเก็บข้อมูล				●	●								
6	เก็บข้อมูลก่อนทำกิจกรรมทดลอง					●	●	●						
7	กลุ่มอาสาสมัครทำกิจกรรมทดลอง					●	●	●	●	●				
8	เก็บข้อมูลหลังทำกิจกรรมทดลอง								●	●	●			
9	รวบรวมข้อมูล										●	●		
10	วิเคราะห์และแปลผลการวิจัย											●	●	
11	เผยแพร่ผลงานวิจัย												●	รายงานฉบับสมบูรณ์

15. แหล่งที่มาของทุน

หลังจากโครงการวิจัยผ่านการรับรองจริยธรรมแล้ว ข้าพเจ้าจะดำเนินการขอรับทุนสนับสนุน

- ✔ จากกองทุนวิจัยมหาวิทยาลัยนวมินทราธิราช
- ☐ ทุนบริษัทยา ชื่อ..
- ☐ ทุนส่วนตัว
- ☐ แหล่งทุนอื่น ๆ ระบุ

16. ค่าตอบแทนให้แก่อาสาสมัคร (ค่าเดินทาง/ค่าเสียเวลา)

มีค่าตอบแทนในการเข้าร่วมวิจัยรายละ 1400 บาทโดยให้ในรูปแบบของบัตรกำนัลออนไลน์ (E-voucher) ในการซื้อหนังสือจากร้าน SE:ED เป็นเงิน 700 บาทหลังจากทำกิจกรรมครบ 3 ครั้ง และ 700 บาท หลังทำกิจกรรมครบ 6 ครั้ง โดยจะส่งให้ผู้ปกครองผ่านทาง Line Application

17. การมีผลประโยชน์ทับซ้อน (Declare Conflict of Interest)

คณะผู้วิจัย ไม่มีผลประโยชน์ทับซ้อนในงานวิจัยนี้

18. เอกสารอ้างอิง

1. Early childhood development [Internet]. UNICEF Thailand. [cited 2021Oct18]. Available from: https://www.unicef.org/thailand/what-we-do/early-childhood-development

2. Halfon N, Shulman E, Hochstein M. Brain development in early childhood. Los Angeles: UCLA Center for Healthier Children, Families and Communities; 2001.

3. Daelmans B, Darmstadt GL, Lombardi J, Black MM, Britto PR, Lye S, et al. Early childhood development: The Foundation of Sustainable Development. The Lancet. 2017;389(10064):9–11.

สำนักงานคณะกรรมการพิจารณาจริยธรรมการวิจัย (ตึกเวชศาสตร์ฟื้นฟู ชั้น 5)
คณะแพทยศาสตร์วชิรพยาบาล มหาวิทยาลัยนวมินทราธิราช
681 ถนนสามเสน แขวงวชิรพยาบาล เขตดุสิต กรุงเทพ ฯ 10300
โทรศัพท์ 0-2244-3843

4.Chan M, Lake A, Hansen K. The early years: Silent emergency or unique opportunity? The Lancet. 2017;389(10064):11–3.

5. Begum T. Parental knowledge, attitudes and practices in early childhood development among low income urban parents. Universal Journal of Public Health. 2019;7(5):214–26.

6. Big rise in number of working mothers [Internet]. BBC News. BBC 2017 [cited 2021Oct18]. Available from: https://www.bbc.com/news business-41399493

7. Manganey J. Understanding Underinvolvement: The Educational Decisions of Motivated, Low-SES Parents. Journal of undergraduate research. 2007;:1–28.

8. Park C, Park YR. The conceptual model on smart phone addiction among early childhood. International Journal of Social Science and Humanity. 2014;4(2):147–50.

9. Damen H, Veerman JW, Vermulst AA, Nieuwhoff R, de Meyer RE, Scholte RH. Parental empowerment: Construct validity and reliability of a Dutch Empowerment Questionnaire (EMPO). Journal of Child and Family Studies. 2016;26(2):424–36.

10. Griffith J. Relation of parental involvement, empowerment, and school traits to student academic performance. The Journal of Educational Research. 1996;90(1):33–41.

11. Olin SS, Hoagwood KE, Rodriguez J, Ramos B, Burton G, Penn M, et al. The application of behavior change theory to family-based services: Improving parent empowerment in children's mental health. Journal of Child and Family Studies. 2009;19(4):462–70.

12. Neale D, Pino-Pasternak D. A review of reminiscing in early childhood settings and links to sustained shared thinking. Educational Psychology Review. 2016;29(3):641–65.

13. Siraj-Blatchford I. Conceptualising progression in the pedagogy of play and sustained shared thinking in early childhood education: a Vygotskian perspective. Faculty of Social Sciences - Papers. 2009;1224.

14. Ridgway A, Quiñones G, Li L. Sustained shared thinking in pedagogical play. Early Childhood Pedagogical Play. 2015;:33–45.

15. Zimmerman MA. Psychological empowerment: Issues and illustrations. American Journal of Community Psychology. 1995;23(5):581–99.

16. Cohen, J. (1977). Statistical power analysis for the behavioral sciences (Rev. ed.). Hillsdale, NJ, US, Lawrence Erlbaum Associates, Inc.

17. ตำราการวิจัยทางคลินิก. ขนาดกลุ่มตัวอย่างในการวิจัยทางคลินิก. กรุงเทพฯ: คณะเวชศาสตร;เขตร้อน มหาวิทยาลัยมหิดล, 2554.

สำนักงานคณะกรรมการพิจารณาจริยธรรมการวิจัย (ตึกเวชศาสตร์ฟื้นฟู ชั้น 5)
คณะแพทยศาสตร์วชิรพยาบาล มหาวิทยาลัยนวมินทราธิราช
681 ถนนสามเสน แขวงวชิรพยาบาล เขตดุสิต กรุงเทพ ฯ 10300
โทรศัพท์ 0-2244-3843
