# Supplementary material for: The efficacy of promoting sustained shared thinking through the use of activity books on parental empowerment; A quasi-experimental study
Source: PLoS One. 2025 Jul 18;20(7):e0328537. doi: 10.1371/journal.pone.0328537 (PMC12273987; doi:10.1371/journal.pone.0328537)
Supplement: S1 Protocol — (ZIP) [file pone.0328537.s004.zip › S4 Protocol/4-Trial study Protocol from VIRB (translated).pdf]

Research Project Proposal

Ethics Review Committee, Faculty of Medicine, Vajira Hospital

1. Title

Thai title: ประสิทธิผลของกิจกรรมที่เน้นการคิดร่วมกันอย่างต่อเนื่องในการเสริมพลังให้กับผู้ปกครอง

English title: The impact of sustained shared thinking-based activity on parental empowerment

2. Principal Investigator

2.1 Thai Name: นางสาว กxxxxx ตxxxxx

English Name: Miss Kxxxxx Txxxxx

Position: Assistant Professor

Department: Department of Psychiatry, Faculty of Medicine Vxxxxx

Phone: xxxxxxxxxx, Mobile: xxxxxxxxxx

E-mail: xxxxxx@xxxxxxx

Responsibilities: Research design, ethics submission, funding application, data analysis, results summary, discussion, manuscript drafting.

2.2 Thai Name: นาย วxxxxx พxxxxx

English Name: Mr. Wxxxxx Pxxxxx

Position: Assistant Professor

Department: Department of Psychiatry, Faculty of Medicine Vxxxxx

Phone: xxxxxxxxxx

E-mail: xxxxxx@xxxxxxx

Responsibilities: Results summary, discussion, manuscript drafting, publication submission.

3. Co-Researchers

3.1 Thai Name: นางสาว มxxxxx ศxxxxx

English Name: Mxxxxx Sxxxxx

Position: Founder of Lxxxxx Co., Ltd., an organizational development consultancy company

Department: -

Phone: xxxxxxxxxx

E-mail: xxxxxx@xxxxxxx

Responsibilities: Research design, experimental activity design, manuscript drafting.

3.2 Thai Name: นางสาว นxxxxx ลxxxxx

English Name: Nxxxxx Lxxxxx

Position: Co-founder of Pxxxxx Lxxxxx, a consultancy company for learning design, Lxxxxx Co., Ltd

Department: -

Phone: xxxxxxxxxx

E-mail: xxxxxx@xxxxxxx

Responsibilities: Research design, experimental activity design, manuscript drafting.

3.3 Thai Name: นางสาว คxxxxx ยxxxxx

English Name: Kxxxxx Yxxxxx

Position: Co-founder of Pxxxxx Lxxxxx, a consultancy company for learning design, Lxxxxx Co., Ltd

Department: -

Phone: xxxxxxxxxx

E-mail: xxxxxx@xxxxxxx

Responsibilities: Research design, experimental activity design, manuscript drafting.

4. Name of the Physician in Charge of the Study Participants

Thai Name: นางสาว กxxxxx ฑxxxxx

English Name: Miss Kxxxxx Txxxxx

Department: Department of Psychiatry, Faculty of Medicine Vxxxxx

Phone: xxxxxxxxxx, Mobile: xxxxxxxxxx

Responsibilities: Research design, ethics submission, funding application, data analysis, results summary, discussion, manuscript drafting.

5. Significance and Background of the Problem

The early childhood period, from birth to age six, is crucial for a child's cognitive and emotional development, as it is a time when the brain rapidly develops. Once this period has passed, it is challenging to correct developmental errors.[1] This development heavily depends on social and environmental stimuli, making the family environment particularly significant, as children spend most of their time with their families during this stage.[2] Studies have shown that quality family time can significantly enhance children's academic performance and reduce future behavioral problems. Additionally, developmental support during this phase is linked to a 25% increase in income when children reach adulthood.[3]

However, currently, early childhood does not receive sufficient stimulation from families, leading to missed opportunities for full potential development. A survey found that 43% of children in low- to middle-income countries face this issue,[4] which is due to a combination of factors, including a lack of knowledge and appropriate attitudes toward early childhood upbringing,[5] increased maternal employment outside the home compared to the past, and beliefs that education is the responsibility of schools rather than parents.[6] Some parents even feel they lack the capability to support their child's development.[7] Furthermore, many parents allow their children to spend most of their time on mobile phones,

leading to a higher rate of early childhood mobile addiction.[8] Therefore, promoting effective family time during early childhood is crucial.

One key factor influencing parents' roles in promoting child development is parental empowerment, which refers to the process and psychological outcome where parents feel capable of managing and handling their parenting roles effectively.[9] Previous research indicates that parents who feel empowered perform better in their roles and actively engage in supporting their child's development and education at school.[10] Thus, increasing parental empowerment is a sustainable way to foster children's development.

Our research team has recognized this issue and aims to create activities that enhance parental empowerment to promote children's cognitive and educational development. The focus will be on working parents who have limited time to support their child's development. The activities are based on the principle of “Parents as Agents of Change,” meaning that parents will act as mediators of their children's intellectual development.[11] This approach will primarily rely on the interaction between parents and children. From a review of previous studies, the concept of "Sustained Shared Thinking" (SST) has been identified as suitable for this research.

Sustained Shared Thinking (SST) is a process where two or more individuals engage in brainstorming and exchanging ideas to produce intellectual outcomes, such as problem-solving, processing, and describing.[12] Previous studies have shown that SST is effective for cognitive development in preschool children.[13] Incorporating SST into play makes the activity enjoyable, encouraging children to share their thoughts for a longer period.[14] Therefore, the research team will include SST in play activities that parents can do with their children, using a workbook as a medium because it is convenient and easy to follow for parents. The activities in the workbook will align with the developmental stages of preschool children.

Regarding the assessment of parental empowerment, the research team developed an evaluation tool based on Zimmerman’s theory,[15] which divides empowerment into three components: psychological, interactional, and behavioral. These components are further divided into 12 subcategories, all of which are covered in the questionnaire developed by the research team.

Currently, no research has studied the effectiveness of SST activities in empowering working parents. Thus, the primary objective of this research is to study the effectiveness of SST in empowering parents. Additionally, the secondary objective is to explore the mechanisms that mediate the relationship between SST and parental empowerment, specifically focusing on parents' perception of their child's developmental progress, stimulated by parental involvement. The results of this study could guide sustainable child development in the future.

6. Objectives

6.1 Primary Objective

- To study the effectiveness of activities focused on Sustained Shared Thinking in empowering parents.

6.2 Secondary Objectives

- To study the effectiveness of activities that focus on idea-sharing in enhancing parents' awareness of their child's developmental progress, stimulated by parental involvement.
- To examine the factors affecting the effectiveness of Sustained Shared Thinking activities in empowering parents and enhancing their perception of their child's developmental progress.

7. Research framework

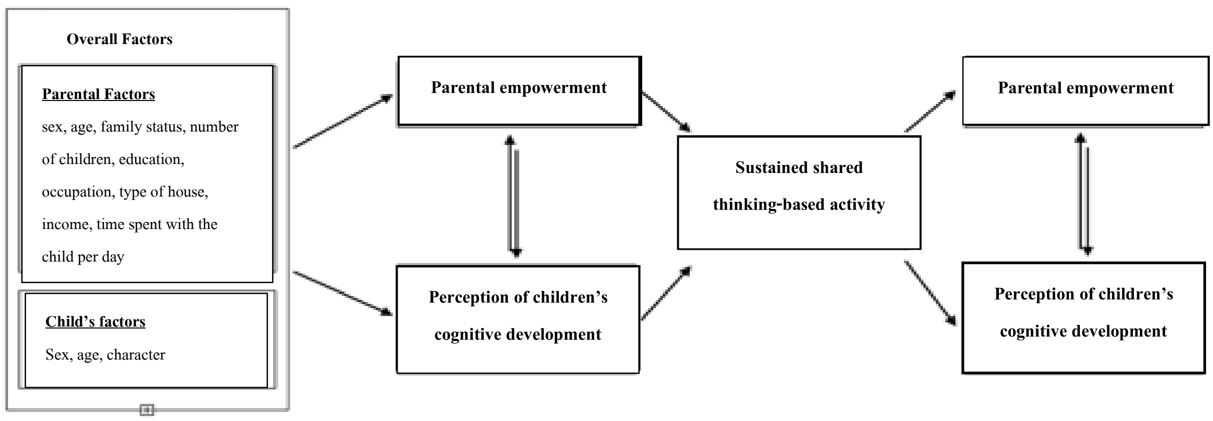

8. Research Design and Methodology

8.1 Research Design

- This is a quasi-experimental study.

8.2 Target Population

- Parents (fathers or mothers) and their children aged 4-6 years.

8.3 Inclusion, Exclusion, and Termination Criteria

Inclusion Criteria:

- Parents aged over 18 years.

- Parents with children aged 4-6 years.
- Parents who live with their children.
- Parents who feel a lack of energy in parenting (based on a questionnaire conducted during the recruitment phase)

Exclusion Criteria:

- Parents with psychiatric disorders.
- Children with neurodevelopmental disorders.
- Children with physical health issues requiring regular medication or frequent medical visits.

Termination Criteria:

- When participants wish to withdraw from the study.

8.4 Population Size

- **Sample Size**:

  This quasi-experimental study aims to compare the means before and after the experiment. Since no prior study is available, there's no reference data for calculating the sample size. The sample size is estimated using G power software version 3.1.9.7, specifying a statistical test as paired t-tests and a power analysis as A priori: Compute required sample size. A significance level α=0.05\alpha = 0.05α=0.05, a test power of 0.9 (90%), and an effect size d=0.5d = 0.5d=0.5 (medium) were used for calculations.[16] The estimated sample size was at least 44 participants. To compensate for potential loss of participants due to follow-up issues or incomplete data (20% attrition), the final sample size is set at 55 participants.[17]

8.5 Variables

Independent Variables:

- **Parental Factors**: Age, gender, education, occupation, income, marital status, household size, number of children, time spent with children.
- **Child Factors**: Age, gender, temperament.
- **Activity**: Sustained shared thinking-based activity.

Dependent Variables:

- **Parental Empowerment**.
- **Perception of children's development**.

8.6 Variable Definitions

- **Sustained Shared Thinking-Based Activity**: Activities conducted between parents and children that involve brainstorming and idea exchange to generate cognitive outcomes, with tools created by the research team as mediators.

- **Parental Empowerment**: The psychological process and outcomes where parents feel capable of managing and handling parenting roles effectively.
- **Perception of Children's Development**: Parents' recognition of their child's progress in thinking, reasoning, and language skills.

8.7 Informed Consent and Recruitment Process

- The study will be advertised through communities. Additionally, announcements will be made in schools with kindergarten levels. Interested participants can contact the research team via telephone and chat application.
- Those interested can scan the QR code and fill in initial information based on inclusion and exclusion criteria. If eligible, the research team will contact the potential participants.
- The researcher will provide information about the study and obtain consent directly from participants, providing research information sheets and consent forms. Verification of participants will be done by taking a photo with their child and sending it to the research team.

8.8 Research Methodology

1. Obtain research approval from the Ethics Review Committee of the Faculty of Medicine Vajira Hospital, Navamindradhiraj University.
2. Develop questionnaires on parental empowerment and perception of children's cognitive development.
3. Conduct a review by experts.
4. Assess reliability and validity.
5. Develop sustained shared thinking activities for the research.
6. Advertise the study through community leaders and contact schools with kindergarten levels to reach more parents.
7. Select participants according to the inclusion and exclusion criteria.
8. Provide research information and request consent via Line (consent will be obtained by filling out the name and pressing the consent button in a Google form). Participants' identities will also be verified by sending a photo with their child to the research team.
9. Collect baseline data, including general information and questionnaires on parental empowerment (15 minutes to complete via Google form on Line or phone interview if necessary).
10. Explain the activity details that parents need to conduct with their children for 6 weeks. Tools and manuals created by the research team will be provided. Activities will be sent weekly, requiring at least one 25-30 minute session per week. Parents must send photos of completed tasks and reflection questions weekly.
11. Collect data after the 3rd and 6th (final) activity sessions, and three months after the final session using questionnaires on parental empowerment and children's cognitive development, as well as open-ended questions about their experience (15-20 minutes to complete).
12. Verify completeness of questionnaires and analyze the data statistically.

8.9 Measurement

1. Questionnaire on general information of participants, covering parents' details (age, gender, education, occupation, income, marital status, household members, number of children, time spent with children) and children's details (age, gender, character).
2. Parental Empowerment Questionnaire.
3. Perception of Children's Cognitive Development Questionnaire.
4. Questionnaire on experience after participating in the study activities.

8.10 Data Collection

Data collection will be carried out by having eligible participants complete questionnaires and assessments. Data will be recorded individually, without displaying any names or information that could identify the participants. Only the research team will have access to this data set, and the team that designed the exercises will not be involved in selecting participants.

Collected data will be checked for completeness, scored according to established criteria, and prepared for statistical analysis.

8.11 Data Analysis

- Descriptive Statistics: Utilizing basic statistics, including percentage, mean, and standard deviation.
- Comparison Analysis: Analyzing parental empowerment scores and perception of children's cognitive development scores before the activities, after the 3rd and 6th sessions, and 3 months post-activities using ANOVA and Linear Mixed Effect Model.
- Qualitative Analysis: The team responsible for designing the exercises will not be involved in data analysis.

8.12 Dummy table

Parent's information	n	percentage
Age		
Sex		
Education		
Occupation		
Income		
Marital status		
Family type		
Number of children		
Time spent with the child		

Child’s information	n	percentage
Age		
Sex		
Character		

Pre-test Questionnaire	Score	SD	Level
parental empowerment			
cognitive development			
literacy skill			

Post-test Questionnaire	Score			SD		
	Week 3	Week 6	3 months	Week 3	Week 6	3 months
parental empowerment						
cognitive development						
literacy skill						

Variables	Pre-test (SD)	Post-test (SD)	t	p-value
parental empowerment				
cognitive development				
literacy skill				

9. Risks and Possible Side Effects

There is a potential psychological risk because participating in activities with children requires physical and emotional effort, along with answering some of the questions may negatively impact mental health. Therefore, participants have the right to refrain from answering or to stop answering the questionnaire, and they can also withdraw from the research. If severe psychological impacts occur, the research team will recommend counseling services with a psychologist or psychiatrist from the Faculty of Medicine at Vajira Hospital.

10. Expected Benefits and Impacts

- The effectiveness of sustained shared thinking activities in empowering parents.
- Development of a questionnaire to measure parental empowerment.
-

11. Ethical Considerations

This research follows the fundamental ethical principles of the Belmont Report:

1. **Respect for Persons**: Providing complete information about the research project to participants without concealing any details. All participants voluntarily agree to participate. Documents related to the research will be kept confidential and accessible only to those involved in the research. Data will be presented in an aggregate form without mentioning the real names of the participants. The research must be approved by an ethics committee before commencing, and all participants or their legal representatives must sign a consent form.
2. **Beneficence**: Participants will be informed of the risks and benefits associated with participating in the research. Participants will also receive guidance on what to do if any abnormalities are detected.
3. **Justice**: Even if individuals do not wish to participate in this research, they will still receive full medical care according to standard treatment guidelines.
4.

12. Research Limitations

Access to research participants will be conducted remotely, primarily through the Line application. This may pose limitations for those who are not familiar with using this type of technology.

13. Potential Challenges and Solutions

This research is conducted at home by the parents themselves, requiring cooperation and honesty from the participants, as it demands a considerable amount of time and effort. Therefore, there may be challenges regarding cooperation. The research team will address this by periodically checking in, providing guidance, and keeping communication channels open for participants to reach out. Additionally, at the end of each activity booklet, there will be reflective questions for parents to complete, and they are required to send photos of these reflections to the research team each week after completing the activities.

14. Research Management and Study Timeline

Step	Details	Month												Output
		1	2	3	4	5	6	7	8	9	10	11	12	
1	Scope problems and objectives	●												
2	Literature review and drafting research proposal	●												
3	Submission for ethical review		●	●										
4	Develop questionnaire and intervention tools				●									Empowerment questionnaire and sustained-shared thinking intervention
5	Collaboration and planning				●	●								
6	Pre-test data collection					●	●	●						
7	Participants conducting experimental activities					●	●	●	●	●				
8	Post-test data collection								●	●	●			
9	Data compilation										●	●		
10	Analyze and Interpret the Research Results											●	●	
11	Disseminate Research Findings												●	Full manuscript

15. Sources of Funding

After the research project receives ethical approval, I will apply for financial support:

☑ From the Research Fund of Navamindradhiraj University.

☐ From a Pharmaceutical Company (Name: ....)

☐ From Personal Funds

☐ From Other Sources (Please Specify)

16. Compensation for Volunteers (Travel/Time Compensation)

Each participant will receive a total compensation of 1,800 Baht for participating in the research, with 300 Baht given after each activity session.

17. Conflict of Interest Declaration

The research team declares no conflict of interest in this study.

18. References

1. Early childhood development [Internet]. UNICEF Thailand. [cited 2021Oct18]. Available from: https://www.unicef.org/thailand/what-we-do/early-childhood-development

2. Halfon N, Shulman E, Hochstein M. Brain development in early childhood. Los Angeles: UCLA Center for Healthier Children, Families and Communities; 2001.

3. Daelmans B, Darmstadt GL, Lombardi J, Black MM, Britto PR, Lye S, et al. Early childhood development: The Foundation of Sustainable Development. The Lancet. 2017;389(10064):9–11.

4.Chan M, Lake A, Hansen K. The early years: Silent emergency or unique opportunity? The Lancet. 2017;389(10064):11–3.

5. Begum T. Parental knowledge, attitudes and practices in early childhood development among low income urban parents. Universal Journal of Public Health. 2019;7(5):214–26.

6. Big rise in number of working mothers [Internet]. BBC News. BBC 2017 [cited 2021Oct18]. Available from:https://www.bbc.com/newsbusiness-41399493

7. Manganey J. Understanding Underinvolvement: The Educational Decisions of Motivated, Low-SES Parents. Journal of undergraduate research. 2007;:1–28.

8. Park C, Park YR. The conceptual model on smart phone addiction among early childhood. International Journal of Social Science and Humanity. 2014;4(2):147–50.

9. Damen H, Veerman JW, Vermulst AA, Nieuwhoff R, de Meyer RE, Scholte RH. Parental empowerment: Construct validity and reliability of a Dutch Empowerment Questionnaire (EMPO). Journal of Child and Family Studies. 2016;26(2):424–36.

10. Griffith J. Relation of parental involvement, empowerment, and school traits to student academic performance. The Journal of Educational Research. 1996;90(1):33–41.

11. Olin SS, Hoagwood KE, Rodriguez J, Ramos B, Burton G, Penn M, et al. The application of behavior change theory to family-based services: Improving parent empowerment in children's mental health. Journal of Child and Family Studies. 2009;19(4):462–70.

12. Neale D, Pino-Pasternak D. A review of reminiscing in early childhood settings and links to sustained shared thinking. Educational Psychology Review. 2016;29(3):641–65.

13. Siraj-Blatchford I. Conceptualising progression in the pedagogy of play and sustained shared thinking in early childhood

education: a Vygotskian perspective. Faculty of Social Sciences - Papers. 2009;1224.
14. Ridgway A, Quiñones G, Li L. Sustained shared thinking in pedagogical play. Early Childhood Pedagogical Play. 2015;:33–45.
15. Zimmerman MA. Psychological empowerment: Issues and illustrations. American Journal of Community Psychology. 1995;23(5):581–99.
16. Cohen, J. (1977). Statistical power analysis for the behavioral sciences (Rev. ed.). Hillsdale, NJ, US, Lawrence Erlbaum Associates, Inc.
17. Overall JE, Shobaki G, Shivakumar C, Steele J. Adjusting sample size for anticipated dropouts in clinical trials. Psychopharmacol Bull. 1998;34(1):25-33.
