# Supplementary material for: The efficacy of promoting sustained shared thinking through the use of activity books on parental empowerment; A quasi-experimental study
Source: PLoS One. 2025 Jul 18;20(7):e0328537. doi: 10.1371/journal.pone.0328537 (PMC12273987; doi:10.1371/journal.pone.0328537)
Supplement: Attachment — Submitted filename: Response to Reviewers.docx [file pone.0328537.s006.docx]

**Response to editor**

Thank you for your detailed feedback and for providing us with the opportunity to revise and resubmit our manuscript to PLOS ONE. We greatly appreciate your insightful comments, and we have provided our responses to each point below.

1. I am reviewing the manuscript thoroughly to ensure it complies with PLOS ONE's style requirements. Let me know if you suggest revising any specific section.
2. I have included the following amended Role of Funder statement in the cover letter as requested: "The funders had no role in study design, data collection and analysis, decision to publish, or preparation of the manuscript."
3. I have carefully reviewed the data and ensured that all personal and identifying information in file <S4 Protocol.zip> has been removed or anonymized in accordance with participant consent and applicable regulations. I have re-uploaded a fully anonymized ensuring that no personal or identifiable information remains. Please let me know if any further adjustments are needed.

**Response to Reviewer#1**

We would like to express our thanks and gratitude for taking your time to improve our manuscript. We truly appreciate your valuable and constructive comments. We have revised our manuscript and listed our responses in the table below. If there are any additional comments, please feel free to let us know.

| **Comment** | **Response** |
| --- | --- |
| Line 119: More information required for the sample size calculations i.e. outcome variable, ratio, one or two-tailed test, family test/statistical test, type of power analysis, etc. | Revised  Line 118-123: We have added “The sample size calculation was performed using the G*Power program. A one-group design with two-tailed test was performed, with parental empowerment specified as the outcome variable. The analysis parameters included an effect size of 0.5, a significance level (α) of 0.05, and a desired statistical power of 0.9. Based on these criteria, the required total sample size was determined to be 44”. |
| Line 143: The English version is to be stated for the empowerment questionnaire. | Revised  Line 180: We have added “English version” at the heading of table 1. |
| Line 156-157: The actual symbol chi-square is to be used. | Revised Line 163-164: We replaced the text with the actual chi-square symbol (χ²). |
| Line 167: The reliability and validity information of the questionnaire is to be provided. | Revised  Line 192-205: We have added “An Exploratory Factor Analysis (EFA) with Varimax rotation was conducted to evaluate the reliability and construct validity of a questionnaire assessing cognitive development and literacy skills. EFA was chosen due to the preliminary nature of the instrument and the sample size (n = 55), with Varimax rotation applied to simplify the interpretation of factor loadings by maximizing the variance of squared loadings across factors. The Kaiser-Meyer-Olkin (KMO) measure of sampling adequacy was 0.72, and Bartlett’s test of sphericity was significant (χ²(45) = 123.56, p < 0.001), confirming the suitability of the data for factor analysis. A two-factor solution was identified, explaining 68.2% of the variance, with item loadings ranging from 0.61 to 0.85 for cognitive development and 0.58 to 0.83 for literacy skills. Cronbach’s alpha coefficients were 0.81 for the cognitive development subscale and 0.79 for the literacy skills subscale, indicating internal consistency and supporting the questionnaire’s reliability and construct validity.”  Line 598-599: We also added ‘Exploratory Factor Analysis on cognitive development and literacy skills questionnaire.’ In supporting information files <S1 Dataset> |
| Line 213: States why the children’s cognitive development and literacy skills were assessed at 3 intervals, not four. | Revised  Line 240-243: To clarify, we have added “To measure change after receiving interventions, children’s cognitive and literacy skills development were evaluated at three intervals: after three weeks, after six weeks, and three months post-intervention. |
| Line 227: The frequency and pattern of missing data/fulfilment of assumptions are to be described before using LOCF. | Revised  Line 257-265: We have addressed missing data patterns and the justification for using LOCF “Demographic analysis of dropout (n = 9) revealed significant differences between the dropout and complete groups in terms of education level (p = 0.002), marital status (p = 0.006), and number of children (p = 0.033), suggesting that certain participant characteristics may be associated with attrition. However, Little’s MCAR (Missing Completely at Random) test yielded a test statistic of 0.0 with a p-value of 1.0, indicating that missingness was completely at random. This result supports the validity of using LOCF, as MCAR ensures that missing data do not introduce systematic bias, making LOCF an appropriate method for imputing missing values in this study.”  Line 436-446: We also acknowledge the limitations for using LOCF “Lastly, the dropout analysis revealed that education level, marital status, and the number of children were significantly different between participants who completed the study and those who dropped out. Specifically, participants with lower educational backgrounds, different marital statuses, and more children were more likely to drop out at the 3-month follow-up. This suggested that certain groups may face structural barriers to continued participation. Given these findings, the use of LOCF remains statistically justifiable but requires cautious interpretation. LOCF assumes stability in parental empowerment scores over time, which may not fully capture the experiences of participants with higher dropout risks. Additionally, targeted interventions, such as improved follow-up strategies for at-risk groups, may help reduce attrition in similar interventions.” |
| Line 229: One or two-tailed test is to be stated. | Revised  Line 255: We have added “employing a two-tailed test” |
| The use of both repeated measures ANOVA and linear mixed-effects model not clear and may seems redundant. State the role for each statistical test. | Revised  Line 253 & 288: Only linear mixed-effects model was carried out. We have deleted “repeated measures ANOVA”. |
| Effect size/CI, post hoc comparison/multiple testing corrections, coefficient, SE, model fit etc are to be presented. | Since we did not use repeated measures ANOVA, therefore, we did not perform these analyses. |
| Per protocol analyses or intent to treat analyses is to be stated. | Revised  Line 256: We have added “intent-to-treat (ITT) analysis” |
| Table 3: for 1- 2 hours per day, 2-4 hours per day etc, is the value 2 in the former represents less than 2 while the latter 2, represents 2 and above? Likewise for others. For income, common categories such as 0-4999 THB, 5000-9999 THB, 10000-14999 THB etc could be utilised. | Revised  We have revised the labels in Table 3 as follows to more accurately reflect the time categories:   - Less than 2 hours per day - 2 to less than 4 hours per day - 4 to less than 6 hours per day - 6 hours or more per day |
| Figure 1: The flowchart looks faint. | We have improved Figure 1 clarity and ensured it meets PLOS requirements by uploading our figure files to the Preflight Analysis and Conversion Engine (PACE), as recommended. The figure has been updated and verified for resolution, file type, and overall quality before resubmission. |
| Figure 2, 3, 4: the figures/legends are to be denoted. | Revised  We have now updated the manuscript to clearly denote the figures and their respective legends for Figures 2, 3, and 4. |
| For parental reflections, the ID code of participants is to be added. | Revised  Line: 330-347: We have added the corresponding participant ID codes to the parental reflections to ensure clarity and proper identification. |

**Response to Reviewer#2**

We would like to express our thanks and gratitude for taking your time to improve our manuscript. We truly appreciate your valuable and constructive comments. We have revised our manuscript and listed our responses in the table below. If there are any additional comments, please feel free to let us know.

| **Comment** | **Response** |
| --- | --- |
| Introduction:  1. Line 77-79- the use of the words "good parent" should be re-written. The word good is a vague and simple word to the point it has little meaning. | Revised  Line 79: We have substituted the word “good” with “effective” to make the point more specific. |
| Methods:  1. There was no control group nor discussion about why the particular evaluation design was chosen. A control group would have improved the rigor and contribution of this study. | Revised Line 432-436: We have added “Thirdly, we selected the methodology of a single-arm, quasi-experimental study for its practical approach and capacity to suggest some level of causality. However, the lack of randomization and absence of a control group restrict this method's ability to establish a definitive causal link between the intervention and the outcome” to the discussion part. |
| 2. What is "child character"? This journal is not specific to family studies, therefore the reader may not know what is meant by the term. This is not a standard demographic factor. Please define this term and how it was measured. | Revised  Line 145-146: We have replaced the term “child character” in the method section with “difficulty of the child to parents (subjective feeling)”, which is already used in the result section. |
| 3. Provide more detail of the empowerment measure that was used. Provide a sentence about how you modified the Zimmerman scale or did you model it and develop this scale in full? This needs to be clarified. | Revised  Line 150-154: To clarify, we have added “The researchers developed this questionnaire in full by using the theory of Zimmerman as a backbone. We formulated questions corresponding to each domain proposed by Zimmerman and conducted confirmatory factor analysis (CFA) to assess whether the questions aligned with their respective domains.” |
| 4. Where did the 160 participants come from to obtain the Cronbach's alpha? This is an important part of the methods but there is little discussion of it. | Revised  Line 171-175: We have added “….based on a sample of 160 participants.  In which, the sample size was determined based on Cronbach’s alpha estimation, assuming an expected Cronbach’s alpha of 0.8, a precision of 0.05, a confidence level of 95%, 12 items, and an anticipated dropout rate of 10%”. |
| 5. It is unclear whether the researchers developed the cognitive and literacy skills measure or modeled them. It appears that they were developed guided by Blooms taxonomy and the Sadiku article. Please provide more detail about this measure. | Revised  Line 182-185: We have provided more details “This questionnaire was listed by our team. It consists of 10 items for parents to evaluate their child's cognitive and language improvement after receiving intervention. The responses are divided into three options: 0 = no improvement, 1 = slight improvement, and 2 = significant improvement.”  Line 192-205: We have added “An Exploratory Factor Analysis (EFA) with Varimax rotation was conducted to evaluate the reliability and construct validity of a questionnaire assessing cognitive development and literacy skills. EFA was chosen due to the preliminary nature of the instrument and the sample size (n = 55), with Varimax rotation applied to simplify the interpretation of factor loadings by maximizing the variance of squared loadings across factors. The Kaiser-Meyer-Olkin (KMO) measure of sampling adequacy was 0.72, and Bartlett’s test of sphericity was significant (χ²(45) = 123.56, p < 0.001), confirming the suitability of the data for factor analysis. A two-factor solution was identified, explaining 68.2% of the variance, with item loadings ranging from 0.61 to 0.85 for cognitive development and 0.58 to 0.83 for literacy skills. Cronbach’s alpha coefficients were 0.81 for the cognitive development subscale and 0.79 for the literacy skills subscale, indicating internal consistency and supporting the questionnaire’s reliability and construct validity.”  Line 598-599: We also added ‘Exploratory Factor Analysis on cognitive development and literacy skills questionnaire.’ In supporting information files <S1 Dataset> |
| 6. The recruitment strategy section is brief. How did you recruit parents through the schools? Fliers? Was there incentives for participation? Was this IRB approved? | Revised  Line 134-135, 139: We have added “The parental empowerment support program was promoted through schools and community networks in Bangkok using fliers that included contact information.” And “Participants received an incentive of 300 THB upon completing each workbook.”  The IRB approved recruitment process and amount of incentive. |
| Results:  No comments. The presentation of the results are appropriate and the figures are helpful. |  |
| Discussion  1. Provide a sentence or two about how the use of activity books can be adapted for other countries where parents may use smart phones for information. Is this still a viable strategy? | Revised  Line 416-418: We have added “Additionally, these workbooks could potentially be converted into a digital format to facilitate easier distribution. However, certain activities that require a physical book would need to be adapted.” |
| 2. The researchers created a new measure of parent empowerment. The researchers briefly cited the use of parent empowerment in the introduction and discussed empowerment as a result of interventions. However, there needs to be added discussion about the measure in relation to those they cited or other measures in the literature. It is context specific, as any empowerment measure should be, but a small paragraph on the state of the parent empowerment measures for parenting interventions would help the reader place this research. | Revised  Line 419-423: We have added “Furthermore, this study also developed the Parental Empowerment Questionnaire, which demonstrated acceptable levels of validity and reliability. Therefore, it can be adapted for use in other research or even translated and reassessed for validity and reliability in different contexts where the measurement of parental empowerment is required”. |
