# Supplementary material for: The efficacy of promoting sustained shared thinking through the use of activity books on parental empowerment; A quasi-experimental study
Source: PLoS One. 2025 Jul 18;20(7):e0328537. doi: 10.1371/journal.pone.0328537 (PMC12273987; doi:10.1371/journal.pone.0328537)
Supplement: Attachment — Submitted filename: Response_to_Reviewers_auresp_2.docx [file pone.0328537.s007.docx]

**Response to academic editor**

Thank you for your detailed feedback and for providing us with the opportunity to revise and resubmit our manuscript to PLOS ONE. We greatly appreciate your insightful comments, and we have provided our responses to each point below.

| **Comment** | **Response** |
| --- | --- |
| Revisions are needed to improve clarity, including replacing vague terms like “good parent” and enhancing overall wording. | Revised   - Line 79-82: we have replaced the term “good parent” and clarified the meaning with “effectively fulfill key parenting responsibilities, such as providing emotional support, setting appropriate boundaries, and engaging in their child’s learning and development” |
| The methods section should provide more detail on the evaluation design, particularly the absence of a control group, and clearly define non-standard terms like “child character.” | - Line 447-451: we have added further discussion about the limitation of the study design as follows, “Thirdly, we selected the methodology of a single-arm, quasi-experimental study for its practical approach and capacity to suggest some level of causality. However, the lack of randomization and absence of a control group restrict this method's ability to establish a definitive causal link between the intervention and the outcome”.   Revised   - Line 149-150: we have replaced the term “child character” with “difficulty of the child to parents (subjective feeling)”. |
| Clarification is required on the development and modification of measurement tools (e.g., empowerment scale, cognitive and literacy skills measures), as well as the origin of the 160 participants used for reliability analysis. | Thank you very much for your suggestions.   - Line 154-161: we have added more details regarding the development of empowerment questionnaire as follows, “The researchers developed this questionnaire in full by using the theory of Zimmerman [20] as a backbone. We formulated questions corresponding to each domain proposed by Zimmerman and conducted confirmatory factor analysis (CFA) to assess whether the questions aligned with their respective domains. Zimmerman categorizes psychological empowerment into 12 subheadings within 3 components, consisting of the intrapersonal component (4 subheadings: item 1-4), the interactional component (5 subheadings: item 5-9), and lastly, the behavioral component (3 subheadings: item 10-12).” - Line 183, 193-206: we have added more information regarding the development of the questionnaire for evaluating children’s development in cognitive and literacy skills as follows; “This questionnaire was listed by our team”. “An Exploratory Factor Analysis (EFA) with Varimax rotation was conducted to evaluate the reliability and construct validity of a questionnaire assessing cognitive development and literacy skills. EFA was chosen due to the preliminary nature of the instrument and the sample size (n = 55) [26], with Varimax rotation applied to simplify the interpretation of factor loadings by maximizing the variance of squared loadings across factors [27]. The Kaiser-Meyer-Olkin (KMO) measure of sampling adequacy was 0.72, and Bartlett’s test of sphericity was significant (χ²(45) = 123.56, p < 0.001), confirming the suitability of the data for factor analysis. A two-factor solution was identified, explaining 68.2% of the variance, with item loadings ranging from 0.61 to 0.85 for cognitive development and 0.58 to 0.83 for literacy skills. Cronbach’s alpha coefficients were 0.81 for the cognitive development subscale and 0.79 for the literacy skills subscale, indicating internal consistency and supporting the questionnaire’s reliability and construct validity”. - Line 175-179: we have added more details regarding the origin of the 160 participants used for the Cronbach's alpha analysis as follows, “the Cronbach’s alpha coefficient for this questionnaire was calculated to be 0.76, based on a sample of 160 participants. In which, the sample size was determined based on Cronbach’s alpha estimation formula, assuming an expected Cronbach’s alpha of 0.8, a precision of 0.05, a confidence level of 95%, 12 items, and an anticipated dropout rate of 10%”. |
| The discussion would benefit from commentary on the use of activity books in digital contexts and a brief comparison of the parent empowerment measure with existing literature to better position the study. | Thank you very much for your valuable suggestion.   - Line 418-431: we have added more discussion about the use of digital version of activity books as follows, “Additionally, these workbooks could potentially be converted into a digital format to facilitate more efficient distribution, given that digital versions incur lower production costs and eliminate shipping expenses, requiring only that parents have access to a suitable device [40]. However, the benefits of using digital versions in terms of promoting parental empowerment remain inconclusive, as existing research presents mixed findings. For example, a study involving preschool children who had no prior exposure to e-books found that those who engaged with e-books for 15 minutes daily over an 8-week period demonstrated greater improvements in emergent literacy skills compared to those who received printed books [41]. In contrast, research involving toddlers indicated that parent-toddler interactions with digital books were characterized by reduced verbalization and collaboration compared to interactions with printed books [42]. These contrasting findings highlight the need for further investigation into how digital and physical formats may differentially influence parental engagement and child development outcomes”. - Line 432-438: we have added our position of the parental empowerment questionnaire as follows, “Furthermore, to our knowledge, this study is the first to develop a parental empowerment questionnaire specifically tested for reliability and validity in biological mothers and fathers of children aged 4 to 6 years. As such, the instrument holds potential for adaptation in future research and may be translated and revalidated in diverse contexts where the assessment of parental empowerment is relevant. This represents an important contribution to the field, addressing a gap in age-specific and role-specific measurement tools”. |

**Response to Reviewer#2**

We would like to express our thanks and gratitude for taking your time to improve our manuscript. We truly appreciate your valuable and constructive comments. We have revised our manuscript and listed our responses in the table below. If there are any additional comments, please feel free to let us know.

| **Comment** | **Response** |
| --- | --- |
| Line 80. Did you mean to write parental or should it be child development? | Thank you very much for your comment.   - Line 81-82: we have rewritten the sentence to make it more understandable. “Empowering parents positively influences the development of their children.” |
| Please describe how you determined eligibility in more detail. Is the General Survey Questions the survey that determined eligibility? If not, when and how did you administer the survey for eligibility? What were the items that were in the eligibility survey? | We have added more details regarding the eligibility assessment process.   - Line 136-143: “The parental empowerment research program was promoted through schools and community networks in Bangkok using fliers that included contact information. Interested and eligible parents contacted the researchers directly through chat application. The screening survey for eligibility was performed via Google Form, asking questions according to inclusion and exclusion criteria. After screening, eligible participants (n=55) provided written informed consent online via Google Form, confirming their agreement by typing their names and checking the consent box. Participants received an incentive of 300 THB upon completing each workbook.” - Line 234-235: “After passing eligibility criteria, all the participants then received demographic questionnaire via Google Form.” - Line 147: we also replace “General Survey questions” with “Demographic questionnaire”, to avoid confusion. |
| Line 163. The pvalue of .333 is not an indicator of a good model fit. It is a modest model fit. Please describe the results of this model more clearly. | Thank you for your comment. We agree that the p-value from the chi-square test alone is not a sufficient indicator of model fit, given its sensitivity to sample size. The revised text now avoids overreliance on the chi-square p-value and provides a more comprehensive assessment of model fit as follows   - Line 167-168: " The model yielded a chi-square (χ²) value of 48.519 was non-significant (p = .333, df = 45)” - Line 173-175: “Taken together, these indicate that the specified confirmatory factor model aligns well with the empirical data and is considered acceptable” |
| Empowerment Questionnaire. Please indicate which items are for which sub-construct of Empowerment. Also, the link between Q12 and Empowerment is unclear. | Revised   - Line158-161: We have revised the manuscript to indicate the mapping of each questionnaire item to its respective sub-construct of empowerment as follows, “Zimmerman categorizes psychological empowerment into 12 subheadings within 3 components, consisting of the intrapersonal component (4 subheadings: item 1-4), the interactional component (5 subheadings: item 5-9), and lastly, the behavioral component (3 subheadings: item 10-12).   Explanation of the link between Q12 and Empowerment.   - Item 12 (“I can manage my emotions and personal life when under stress”) in the empowerment questionnaire falls under the behavioral component, which addresses coping behaviors related to managing stress or adapting to change. The item has a statistically significant standardized loading of 0.30 (p = .002). Although, its loading is somewhat lower than other items in this factor, it still contributes meaningfully to the construct, as reflected in both the CFA output and the structural diagram (see Supporting information files: S1 Dataset: Dataset1) |
